# A comparative analysis of statistical methods to estimate the reproduction number in emerging epidemics with implications for the current COVID-19 pandemic

**DOI:** 10.1093/cid/ciaa1599

**Published:** 2020-10-20

**Authors:** Megan O’Driscoll, Carole Harry, Christl A Donnelly, Anne Cori, Ilaria Dorigatti

**Affiliations:** 1 MRC Centre for Global Infectious Disease Analysis, Department of Infectious Disease Epidemiology, School of Public Health, Imperial College London, London, United Kingdom; 2 Department of Genetics, University of Cambridge, Cambridge, United Kingdom; 3 Mines ParisTech, Paris & Université Paris-Saclay, Orsay, France; 4 Department of Statistics, University of Oxford, Oxford, United Kingdom

**Keywords:** Outbreak analysis, SARS-CoV-2, reproduction number, estimation method comparison, emerging epidemics

## Abstract

**Background:**

As the SARS-CoV-2 pandemic continues its rapid global spread, quantification of local transmission patterns has been, and will continue to be, critical for guiding pandemic response. Understanding the accuracy and limitations of statistical methods to estimate the basic reproduction number, R_0_, in the context of emerging epidemics is therefore vital to ensure appropriate interpretation of results and the subsequent implications for control efforts.

**Methods:**

Using simulated epidemic data we assess the performance of 7 commonly-used statistical methods to estimate R_0_ as they would be applied in a real-time outbreak analysis scenario – fitting to an increasing number of data points over time and with varying levels of random noise in the data. Method comparison was also conducted on empirical outbreak data, using Zika surveillance data from the 2015-2016 epidemic in Latin America and the Caribbean.

**Results:**

We find that most methods considered here frequently over-estimate R_0_ in the early stages of epidemic growth on simulated data, the magnitude of which decreases when fitted to an increasing number of time points. This trend of decreasing bias over time can easily lead to incorrect conclusions about the course of the epidemic or the need for control efforts.

**Conclusions:**

We show that true changes in pathogen transmissibility can be difficult to disentangle from changes in methodological accuracy and precision in the early stages of epidemic growth, particularly for data with significant over-dispersion. As localised epidemics of SARS-CoV-2 take hold around the globe, awareness of this trend will be important for appropriately cautious interpretation of results and subsequent guidance for control efforts.

## Keywords

Outbreak analysis; SARS-CoV-2; reproduction number; estimation method comparison; emerging epidemics

Introduction

The reproduction number, R, is a key epidemiological parameter that quantifies the average number of new infections caused by a single infected individual. When a pathogen emerges in an entirely susceptible population this parameter is referred to as the basic reproduction number, $R_0$. When some population-level immunity exists, the parameter is referred to as the effective reproduction number, $R_e$. These reproduction numbers provide valuable information about a pathogen's potential for spread in a population and the associated implications for control efforts (1–3). Reproduction number estimates for any one pathogen are time and context specific, with factors such as contact patterns, population immunity, and behavioral change contributing to variability in these estimates (4, 5). For these reasons, the reproduction number is usually monitored over time to track the progress of an outbreak. The instantaneous reproduction number, $R_t$, estimates the average number of secondary infections generated by an infected individual at time t and sequential estimates over the course of an outbreak can provide valuable insights into the need for interventions and/or the effectiveness of control programmes already in place. In the case of the Walling and Teunis method, $R_t$ is used to refer to the case reproduction number, which quantifies transmission within a cohort of individuals, i.e. individuals with the same date of infection or symptom onset, providing a retrospective quantification of transmissibility.

Numerous mathematical and statistical methods have been developed to estimate the reproduction number of an emerging pathogen (6–12). The choice of method to be used largely depends on the available data, with more data typically allowing the parameterization and use of more complex methods. However, in the early stages of an outbreak, epidemiological data are often sparse and highly uncertain. In the case of emerging pathogens, data can be particularly limited, as surveillance systems may be unprepared for the detection and reporting of a new pathogen. In addition, limited understanding of the dynamics of a new pathogen can often inhibit the parameterization and use of more complex transmission models. This has particularly been the case for SARS-CoV-2, which was first reported in China in December 2019 and has since reached more than 235 countries and territories, as of October 2020 (13). Though new evidence is rapidly emerging on aspects of transmission dynamics such as generation intervals and the proportion of asymptomatic infections (14), uncertainties remain as to the duration of immunity, the role of seasonality and the effects of immunological cross-reactivity with endemic human coronaviruses. Incorporating such uncertainties into mechanistic models of transmission can prove challenging in early outbreak contexts, where statistical methods are often the only available tool to infer the level of transmission from a limited amount of data, such as time series of reported case numbers. Quantifying local transmission patterns of SARS-CoV-2 has been, and will continue to be, critical for guiding the pandemic response (15–18). Understanding the accuracy and limitations of these methods in the context of real-time outbreak analysis scenarios is crucial to optimally inform outbreak response activities, including the need for new control efforts or the relaxation of efforts already in place.

Here, we conduct a comparative performance analysis of 7 commonly-used statistical methods to estimate the reproduction number at sequential time points during the early stages of an emerging epidemic, in line with a real-time outbreak analysis scenario.

## Methods

Incidence data were simulated using a deterministic compartmental SEIR (susceptible, exposed, infectious, recovered) model of transmission, assuming closed populations of variable sizes and homogeneous mixing, random time-constant basic reproduction numbers, $R_0$, a variable number of infections seeding the epidemics, under-reporting and different levels of stochasticity in the reporting and infection process (details are given in the supplementary methods S1.

Estimating an average, fixed $R_0$, we assessed the performance of each statistical method by fitting to the simulated epidemic data at sequential time points in the epidemic growth phase, in line with a real-time outbreak analysis scenario. A detailed description of each of the 7 statistical methods is given in supplementary methods S2. A fixed gamma distribution for the generation time interval with mean of 20 days and standard deviation of 7.4 days was assumed to be known for all methods (29). We initiated the analysis 6 weeks into each epidemic, fitting to the first 6, 9, 12, 15, etc., weeks (approximating to 2, 3, 4, 5, etc., generation times), up to the peak of the epidemic, simply defined as the week with the maximum number of cases from the entire epidemic curve. The performance of each method was assessed at each of these stages, with methods being fit to an increasing number of data points. We used a number of metrics to assess method performance: bias, calculated as the absolute difference between the estimated and true $R_0$ values; coverage, calculated as the proportion of times the estimated 95% confidence intervals included the true $R_0$ value; Pearson correlation coefficient (PCC); width of 95% confidence intervals; and the root mean square error (RMSE). To ensure a systematic comparison of performance we only compared the results from sections of the epidemic time series where all methods were able to produce estimates of $R_0$.

We applied each method to weekly case-notification data from the 2015-2016 Zika epidemic in Latin America and the Caribbean. National-level data were available for 37 countries, 3 of which reported their peak incidence within 6 weeks of the first reported case and were subsequently removed from analysis. In the same manner as the simulated data, we applied each of the 7 methods to an increasing number of data points (using the first 6, 9, 12, 15, etc., weeks), up to the peak of the epidemic.

## Results

Biases in estimates of $R_0$ (estimated $R_0$ – actual $R_0$) when fitted to an increasing number of time points in the case time series (6, 9, 12 and 15 weeks) of simulated data are shown in Figure 1 and Supplementary Figure S2 for the scenario of data with no added noise (i.e. assuming that a constant proportion of infected individuals are detected and reported as cases). Estimates of $R_0$ were frequently over-estimated at all time points assessed, the magnitude of which was generally greater for higher values of actual $R_0$ (Figure S2). As expected, higher values of $R_0$ used for data simulation generally resulted in epidemics that peaked at earlier time points, subsequently resulting in a reduction in the number of epidemic simulations where the growth phase continues beyond 9, 12, 15, etc., weeks, and an under-representation of high $R_0$ simulations at the later epidemic stages assessed (Figure S2). To ensure a systematic analysis of methodological performance at sequential time points in the epidemic time series, assessment of performance was restricted to epidemic simulations that peaked at or after 15 weeks, highlighted by the blue points in Figure S2.

Average bias between estimated and actual $R_0$ values was highest in the earliest stages assessed for all methods considered (Figure 1 and S3), with mean absolute differences between estimated and actual $R_0$ values ranging from 0.68 (Bettencourt and Ribeiro) to 1.29 (EpiEstim) when fitting to the first 6 weeks of data (approximately 2 disease generations) of the epidemic growth phase with no added noise. Bias decreased substantially when fitting to an increasing number of time points and ranged from 0.32 (Poisson and maximum-likelihood exponential growth) to 0.48 (Bettencourt and Ribeiro) at 15 weeks (approximately 5 disease generations) in the scenario of no random noise. Density distributions of bias from the scenarios of simulated epidemic data with added noise are shown in Figure S6. For epidemic simulations with Poisson noise, mean absolute bias ranged from 0.60 (Bettencourt and Ribeiro) to 1.65 (EpiEstim) at 6 weeks and from 0.34 (Poisson and maximum-likelihood exponential growth) to 0.46 (Bettencourt and Ribeiro) at 15 weeks. Similarly, for epidemic simulations with negative binomial noise, mean absolute bias ranged from 0.71 (Bettencourt and Ribeiro) to 2.52 (Wallinga and Teunis) at 6 weeks and from 0.29 (linear exponential growth) to 0.82 (Wallinga and Teunis) at 15 weeks. The relationship between estimated and actual $R_0$ by method and time point are shown in Figures S3-S5 for the scenarios of data with no noise, Poisson noise and negative binomial noise, respectively.

Coverage of actual $R_0$ values, i.e. the proportion of simulations in which the 95% confidence interval contained the actual value of $R_0$, decreased when fitted to an increasing number of time points in the case time series for all methods assessed and in all noise scenarios (Figure 2). This decrease in coverage of actual $R_0$ values corresponds to reduced uncertainty, i.e. width of the 95% confidence intervals, surrounding the estimates of all methods when fitted to an increasing number of time points, as shown in Figure 2. In the scenario of data with no noise, coverage ranged from 46% (Bettencourt & Ribeiro) to 96% (Wallinga and Teunis) at 6 weeks and from 25% (linear exponential growth) to 75% (Wallinga and Teunis) at 15 weeks. When fitted to simulations with Poisson random noise, coverage ranged from 54% (EpiEstim) to 92% (Wallinga and Teunis) at 6 weeks and from 50% (Poisson and maximum-likelihood exponential growth) to 74% (Wallinga and Teunis) at 15 weeks. For simulations with negative binomial random noise, coverage ranged from 51% (EpiEstim) to 98% (linear exponential growth) at 6 weeks and from 45% (Poisson and maximum likelihood exponential growth) to 90% (linear exponential growth) at 15 weeks. A significant increase in the levels of uncertainty in the presence of increasing random noise was observed for each of the exponential growth methods (Figure 2). This increase in uncertainty was most notable for the linear exponential growth method, with average confidence interval widths (averaged across all time points assessed) of 0.87, 2.70 and 4.13 when fitted to data with no noise, mild noise (Poisson) and high noise (negative binomial), respectively. Only minor increases in uncertainty were observed for the EpiEstim (1.55, 1.61, 1.73), White and Pagano (2.24, 2.33, 2.52) and Bettencourt and Ribeiro methods (1.35, 1.76, 1.84). No significant differences in levels of uncertainty were observed for the Walling and Teunis method with the addition of random noise to the data (3.74, 3.49, 3.48).

The Pearson correlation coefficient between estimated and actual $R_0$ values was close to 1 for all methods in the scenario of data with no added noise at all time points, though a slight reduction in this correlation was observed at 15 weeks for all methods except linear exponential growth (Figure 2). Reduced correlation between estimated and actual $R_0$ was observed for the scenarios of data with random noise at all stages of the epidemic, the most significant reduction of which was observed at the earliest time point assessed (6 weeks). The RMSE decreased when fitted to increasing numbers of data points for all methods considered and in all data noise scenarios. Trends in method-specific RMSE were similar for data with no noise and Poisson noise, though RMSEs increased significantly in the presence of negative binomial noise for all methods, particularly in the earlier epidemic stages assessed.

Figure 3 shows the $R_0$ estimates obtained from fitting each of the 7 methods to empirical outbreak data, with the examples of French Guyana, Martinique, Puerto Rico and the US Virgin Islands highlighting empirical outbreaks where estimates of $R_0$ decreased when fitting to an increasing number of time points in the case time series during the phase of early epidemic growth. The estimates obtained for all Latin American and the Caribbean countries are shown in Figure S7. $R_0$ estimates were generally higher in the early stages of the epidemic for all methods assessed, with estimates decreasing gradually over time. Confidence interval widths were also observed to generally decline when fitted to an increasing number of datapoints. Frequent inconsistencies between estimates of $R_0$ produced by different methods on the same data were observed, particularly during the early growth phase of the epidemics, e.g. French Guyana, Martinique and Brazil.

Density distributions of bias in $R_0$ estimates when fitting to epidemic simulations using different generation time distributions, are shown in Figure 4. Trends in bias over time were similar when fitted to simulations for Zika and Ebola generation time distributions. Larger average bias was observed for most methods and time points when fitted to simulations for the SARS generation time distribution. Bias in estimates of $R_0$ when the mean generation time is mis-specified are shown in Figure S8. Overestimation of the mean generation interval resulted in overestimation of $R_0$ estimates, while underestimation of the mean generation interval resulted in an underestimation of $R_0$ estimates, as compared to results where the mean generation interval was correctly specified. Further sensitivity analysis regarding the assumed reporting fraction showed reduced bias at the earliest fitting stage (6 weeks) when assuming 100% of infection reporting, as compared to assuming only 20% of infections result in reported cases (Figure S9).

## Discussion

In the early stages of an outbreak response, efforts are often dedicated to estimating pathogen transmissibility in order to provide information on the potential for spread in the current population and to inform the type and scale of interventions required for control (19, 20). Our results show variable accuracy of $R_0$ estimates obtained, both between individual methods as well as across different stages of the early growth phase of the epidemic. Estimates of $R_0$ obtained for the earliest stages of the epidemic assessed in this analysis (i.e. at 6 and 9 weeks, corresponding to approximately 2 and 3 generation time intervals respectively) were associated with larger bias and uncertainty for all methods assessed. Using simulated epidemic data, we found that estimates of $R_0$ frequently overestimated the actual $R_0$ value used in the simulation process, particularly in the early fitting stages even when the true case time series is observed. Estimates of $R_0$ became increasingly accurate for all methods when fitted to an increasing number of weeks in the case time series. Higher average absolute bias was observed for all methods when fitting to simulated data with added random noise, particularly in the earlier stages assessed (Figure S6).

Whilst the over-estimation of $R_0$ in the early stages of an epidemic may be preferable to underestimation for purposes relating to the planning of outbreak response activities, reductions in the magnitude of this over-estimation over time can potentially lead to misconceptions about trends in transmissibility. The trend of decreasing average $R_0$ estimated in the early stages of epidemic growth was also observed for some countries when fitting to national case surveillance data from the 2015-2016 Zika epidemic in Latin America and the Caribbean (e.g. French Guyana, Martinique, Puerto Rico and the US Virgin Islands as shown in Figure 3). The limited efficacy of existing interventions against vector-borne pathogens such as Zika suggest the possibility that methodological bias could explain these trends rather than reductions in transmission at that time. In addition, early analysis of the SARS-CoV-2 epidemic in China estimated a declining trend in the reproduction number from 7.93 (95% CI: 5.00-12.00) on the 29th December 2019 to 2.60 (95% CI: 0.57-5.17) on the 18th January 2020 using the Wallinga and Teunis method, attributing this reduction to the effectiveness of prevention and control measures taken at that time (21). However, the largest decrease in $R_t$ estimated by Liu et al., was observed between the 29th of December and the 2nd of January 2020, prior to the implementation of significant interventions, from which point the estimates of R largely stabilised for the remainder of the period of estimation (21). Awareness of this bias is crucial for critical evaluation of the transmissibility estimates obtained in the early epidemic stages and for accurately interpreting subsequent implications for control (22).

In the current context of the SARS-CoV-2 pandemic, caution is required when using these methods for the estimation of $R_0$. As countries expand surveillance systems to better manage the pandemic, the assumption of constant reporting of cases implicit in these methods likely do not hold true until the capacity of the surveillance system has stabilised, and a consistent case definition is applied. In these contexts, whilst testing capacity is growing, the use of hospital admission or death data, where available, may be preferable for inferring $R_0$ if the reporting of these data is believed to be constant in time. Delays in the time from infection to hospitalisation and/or death, however, result in significant lags between when transmission events occur and when they can be quantified. Regardless of the metric used for estimation of $R_0$, we urge caution when interpreting short-term fluctuations in these estimates as variable data quality and methodological accuracy may play a role in these trends.

There are a number of limitations in our analysis, including the simplicity inherent in the assumptions of homogenous mixing in fully susceptible and closed populations that were used to simulate the case data. These assumptions do not reproduce the sub-exponential growth dynamics that can occur in empirical epidemics as driven by complex spatial structures, local contact networks and other socio-behavioural factors not accounted for in this analysis (24, 25). Many methods exist that account for additional complexities in the epidemic process such as simultaneous estimation of R and the serial interval, the use of household or contact tracing data or the importation of cases from external populations (26-28). The results of this analysis are based on weekly epidemic case-time series, which may not fully reflect results obtained when daily case reports are available. However, whilst the larger number of datapoints provided by daily time series may potentially improve methodological performance in the early epidemic stages, daily case counts may be subject to larger overdispersion and substantial day-of-the-week effects. In addition, noise was added to the case time series in the form of random errors to reflect stochasticity in the infection and reporting processes, inherently assuming the noise to be random. Systematic forms of noise/bias, such as reporting delays, changes in health-seeking behaviour or case definitions are not accounted for in this analysis. Here, we show that $R_0$ estimates frequently overestimate the actual $R_0$ values even in the absence of noise, when we assume that a constant proportion of infections is accurately detected and reported, when the true generation time is known, and when the assumption of exponential growth is met (both in the simulation model and in the estimation method). Our finding that the bias in $R_0$ estimates persists even in the absence of under-reporting in the simulation stage, suggests that the bias may be attributable to methodological differences, which warrants further research. The difference in bias observed at the earliest fitting stage under different assumptions of reporting rates can be explained by missing infections at the beginning of the case time series when under-reporting is present. Delayed detection and reporting of cases at the beginning of an epidemic is common, and particularly so for newly-emerged pathogens and caution should be taken when considering the first reported cases which for many pathogens are unlikely to be the first acquired infections.

In this analysis we highlight the varying strengths and limitations of 7 commonly-used statistical methods for estimating $R_0$ in emerging epidemics. We show how the performance of these methods can vary over time when fit to increasing amounts of data, in line with a real-time outbreak analysis scenario, and demonstrate the sensitivity of methodological performance to varying levels of random noise in the data. This has important implications for the ongoing SARS-CoV-2 pandemic as we show that true changes in transmissibility in the early stages of epidemic growth may be difficult to disentangle from changes in methodological accuracy and precision, particularly for data with significant overdispersion. Cautious interpretation is warranted when using these methods to infer transmission patterns in the early stages of epidemic growth, as apparent declines in $R_0$ estimates may be mis-attributed to the effectiveness of control efforts and lead to incorrect conclusions about the course of the epidemic. Several generations of pathogen transmission may need to be observed for accurate $R_0$ estimation by these methods. An awareness of this trend in bias over time is crucial for appropriate interpretation of $R_0$ estimates and any subsequent implications for the planning of outbreak response activities.

Acknowledgments

We thank Dr Zulma Cucunubá for sharing an updated version of the Zika incidence data from Latin America and the Caribbean (26) and Dr Kris Parag for his thoughtful comments on the analysis.

Funding

This work is jointly funded by the UK Medical Research Council (MRC) and the UK Foreign, Commonwealth & Development Office (FCDO)under the MRC/DFID Concordat agreement and is also part of the EDCTP2 programme supported by the European Union (MO, CAD, AC, ID); the Fondation Mathématiques Jacques Hadamard (CH); the Imperial College Undergraduate Research Opportunity Programme (CH); the Imperial College Junior Research Fellowship, Wellcome Trust and the Royal Society [grant 213494/Z/18/Z] (ID).

Potential Conflicts of Interest

A.C. reports grants from Medical Research Council, NIHR, and USAID, outside the submitted work. I.D. reports grants from Wellcome Trust, Medical Research Council, Janssen R&D, and Natural Environment Research Council, outside the submitted work. C.D. reports grants from UK Medical Research Council and NIHR, outside the submitted work.  All other authors have no potential conflicts.

Data Statement

The data and R code necessary to reproduce this analysis are available on GitHub: https://github.com/meganodris/R0-methods-comparison

## FIGURE LEGENDS

**Figure 1. Density distributions of bias in $R_0$ estimates (estimated $R_0$ – actual $R_0$) obtained when fitting to the case time series on simulated data, without noise, by method and time point (in weeks), using only results from simulations that peaked at or after 15 weeks (N=145).** Columns represent the number of datapoints (weeks) each method was fitted to in the case time series (6,9,12,15 weeks, approximating to 2,3,4,5 generation times), and colours represent the method. Black dashed lines highlight the ideal bias value of zero and coloured lines represent method-specific values of median bias. Method abbreviations: Linear exponential growth rate method (EG_Lin); Poisson exponential growth rate method (EG_P; maximum likelihood exponential growth rate method (EG_MLE); White and Pagano method (WP), Wallinga and Teunis (WT), Bettencourt and Ribeiro (BR).

**Figure 2. Comparative analysis of the performance of the 6 methods at different stages of the epidemic.** Columns represent the three data noise scenarios explored (no noise, Poisson noise and negative binomial noise). Rows represent different performance metrics: Absolute bias (the absolute average difference between estimated and true $R_0$ values), Uncertainty (95% confidence interval width), Coverage (proportion of times in which the true $R_0$ value is within the estimated 95% confidence intervals), Pearson correlation coefficient (PCC), and root mean squared error (RMSE). Method abbreviations: Linear exponential growth rate method (EG_Lin); Poisson exponential growth rate method (EG_P); maximum likelihood exponential growth rate method (EG_MLE); White and Pagano method (WP), Wallinga and Teunis (WT), Bettencourt and Ribeiro (BR).

**Figure 3. $R_0$ estimates obtained from each of the six methods fitted at different stages of the 2015-2016 Zika epidemics in French Guyana, Martinique, Puerto Rico, and the US Virgin Islands**. The top panel for each country shows the time series of reported Zika cases, with dashed lines showing the different stages at which each method was fitted to the data (first 6, 9, 12, etc., weeks) up to the peak of the epidemic, marked by the black line. The bottom panel for each country shows the mean and 95% confidence intervals of the $R_0$ estimates produced with each method fitted to each time series. Method abbreviations: Linear exponential growth rate method (EG_Lin); Poisson exponential growth rate method (EG_P); maximum likelihood exponential growth rate method (EG_MLE); White and Pagano method (WP), Wallinga and Teunis (WT), Bettencourt and Ribeiro (BR).

**Figure 4. Density distribution of bias in $R_0$ estimates (estimated $R_0$ – actual $R_0$) obtained when fitting to the case time series of simulated data, without noise, by method and time point (in approximate generations) using only results from simulations that peaked at or after 15 weeks.** Columns represent the approximate number of disease generations fitted to in the case time series, and colours represent the method. Black dashed lines highlight the ideal bias value of zero and coloured lines represent method-specific values of median bias. The generation time distribution used, both for data simulation and method fitting, is shown on the y-axis. Mean and standard deviation in days for generation time distributions used: Zika (20±7.4); Ebola (16±9.3); SARS (8±3.8). Method abbreviations: Linear exponential growth rate method (EG_Lin); Poisson exponential growth rate method (EG_P); maximum likelihood exponential growth rate method (EG_MLE); White and Pagano method (WP), Wallinga and Teunis (WT), Bettencourt and Ribeiro (BR).

Figure 1

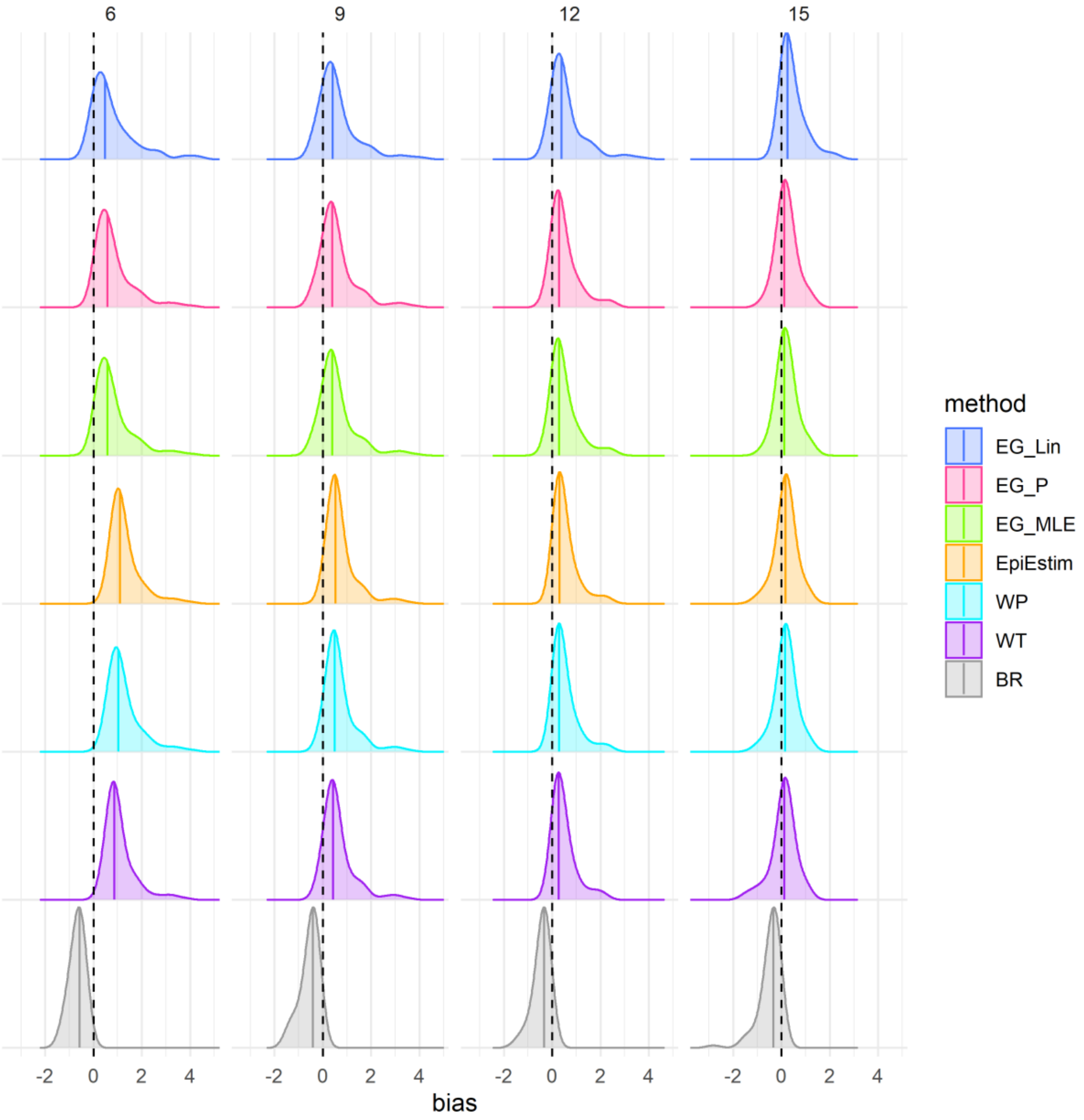

Figure 2

None
Poisson
NegBinom
Bias
Uncertainty
Coverage
PCC
RMSE
Performance
Number of weeks
method
EG_Lin
EG_P
EG_MLE
EpiEstim
WP
WT
BR

Acc

Figure 3

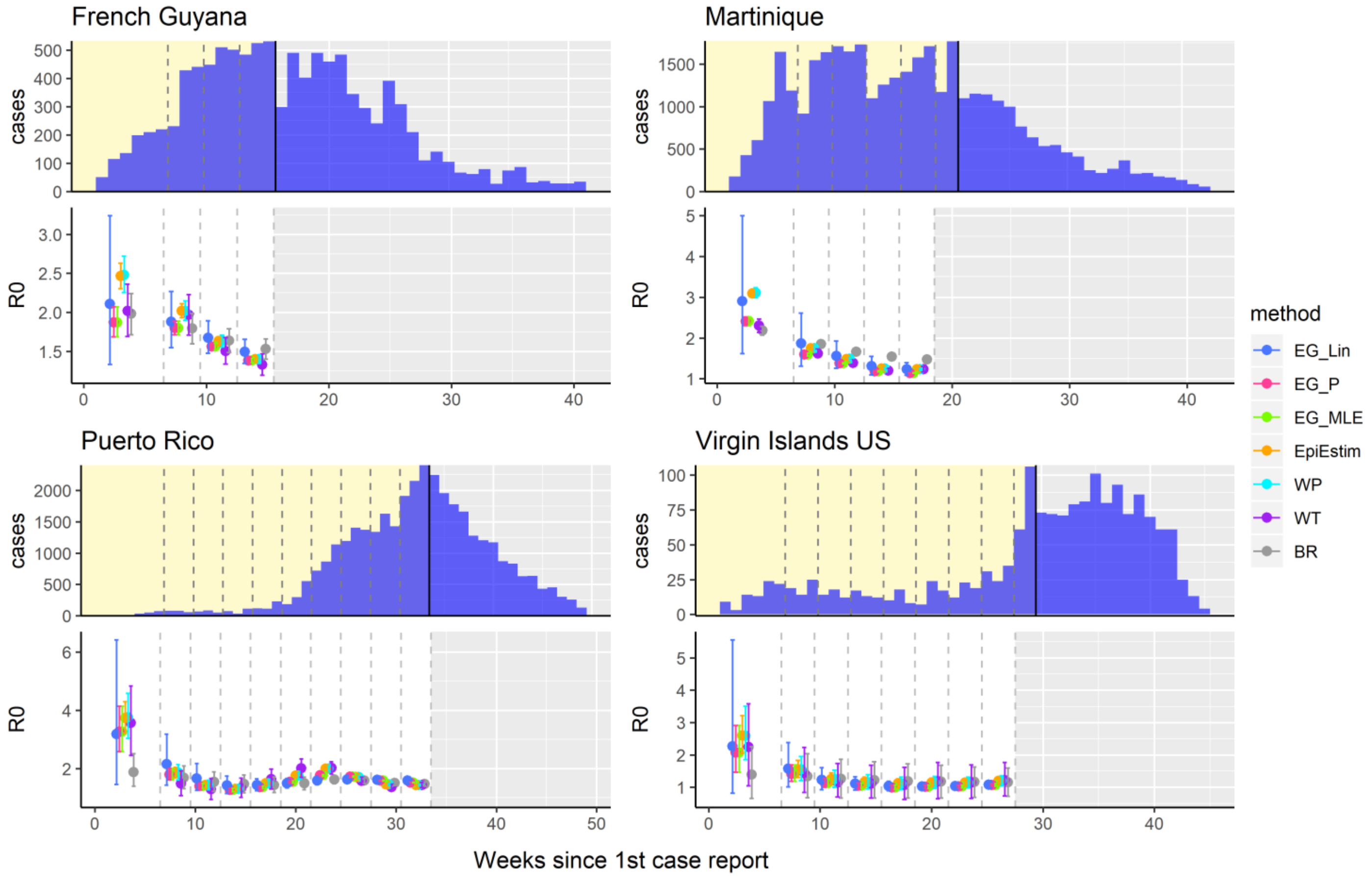

Figure 4

2
3
4
5
Zika
Ebola
SARS
EG_Lin
EG_P
EG_MLE
EpiEstim
WP
WT
BR
-2
0
2
4
bias
