# Supplementary material for: A Comparative Analysis of Statistical Methods to Estimate the Reproduction Number in Emerging Epidemics, With Implications for the Current Coronavirus Disease 2019 (COVID-19) Pandemic
Source: Clin Infect Dis. 2020 Oct 20;73(1):e215–23. doi: 10.1093/cid/ciaa1599 (PMC7665402; doi:10.1093/cid/ciaa1599)
Supplement: ciaa1599_suppl_Supplementary_Materials [file ciaa1599_suppl_supplementary_materials.docx]

### **Supplementary Information**

*Supplementary Methods S1. Data simulation*

Values of R_0_ were randomly selected from a truncated normal distribution with a mean of 2, standard deviation of 2, truncated between 1.2 and 8 (Figure S1 in supplementary information). Using the example of Zika, we assumed average incubation and infectious periods of 14 and 6 days, respectively, for data simulation, giving an average generation time of 20 days, standard deviation 7.4, which we assumed to be known for model fitting (Figure S1 in supplementary information) (29). We assumed there was no transmission before the onset of symptoms. We also assumed entirely susceptible closed populations with randomly selected sizes ranging in multiples of 10, from 10^3^ to 10^6^, and assumed homogenous mixing in the population. The epidemic was seeded with a random number of between 1 and 5 initially infected individuals. Daily incidence data were simulated for a period of 2 years which was then aggregated to weekly data for consistency with the empirical data reported during the 2015-2016 Zika epidemic in Latin America and the Caribbean. We assumed that 20% of all infections resulted in reported case-notifications which was constant over time. 250 unmitigated epidemic simulations were generated, and from each trajectory we generated 3 different time series assuming different levels of random noise. To represent stochasticity in the reporting and infection processes Poisson (mild noise) and negative binomial (high noise, dispersion parameter=3) distributed random errors were separately added to the simulated epidemic.

*Supplementary Methods S2. Statistical methods*

***1. Exponential Growth methods***

The exponential growth rate, *r*, is the per capita change in the number of new cases per unit of time. This growth rate can be linked to the basic reproduction number, *R_0_*, through the moment generating function of the generation time distribution, *g(a)*, (30) as shown in equation 1:

_
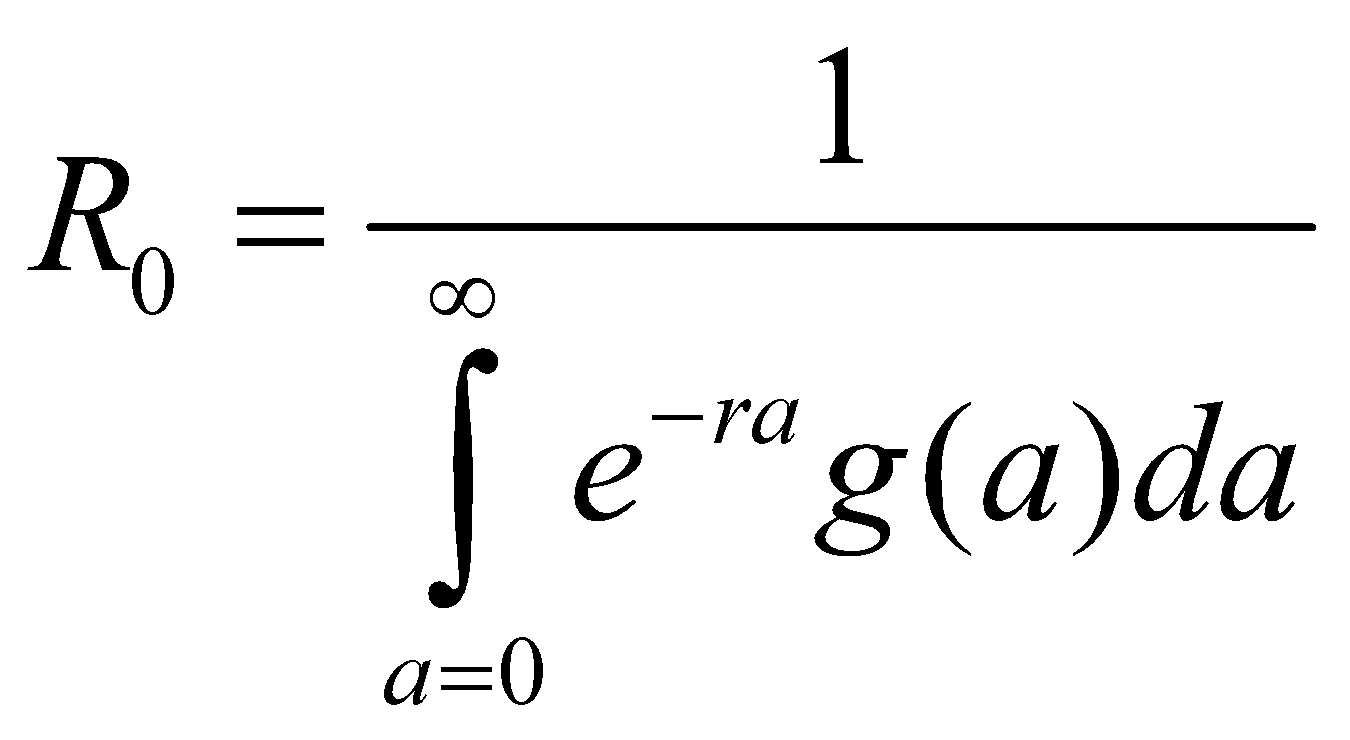
_

[1]

As the name suggests, this method assumes the epidemic to be growing at an exponential rate and with a known generation time distribution, *g(a)*. This method can be applied to the case time series using Poisson regression which we denote as “EG_P”, or on the logarithm of the case time series using linear regression, denoted “EG_Lin”. We apply both Poisson and linear regressions in this analysis. The 95% confidence interval around the central R_0_ estimates were derived by taking the bounds of the 95% confidence interval for the estimate of r, which were transformed to R_0_ values as in equation 1.

In addition, we applied a maximum likelihood version of the method to estimate both the exponential growth rate *r* and *I_0_*, the initial number of infected individuals, assuming that incidence at time *t*, I_t_, is Poisson distributed with mean _
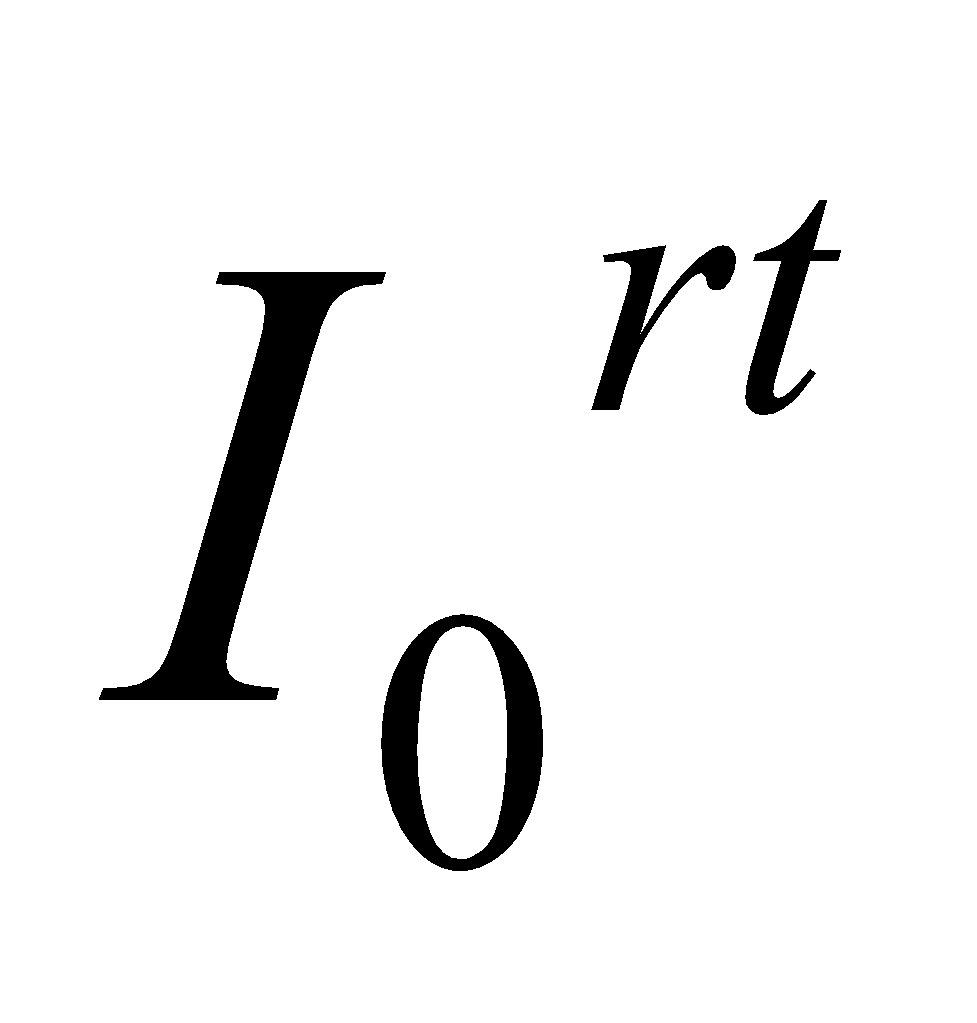
_, which we denote as “EG_MLE”.

***2. White and Pagano maximum likelihood method***

The White and Pagano method uses a maximum likelihood framework with the log-likelihood (LL) shown in equation 2. The mean case incidence, *µ_t_,* is defined as shown in equation 3, where *I_t_* denotes the observed incidence at time *t* and *g(s)* denotes the generation interval distribution.

_
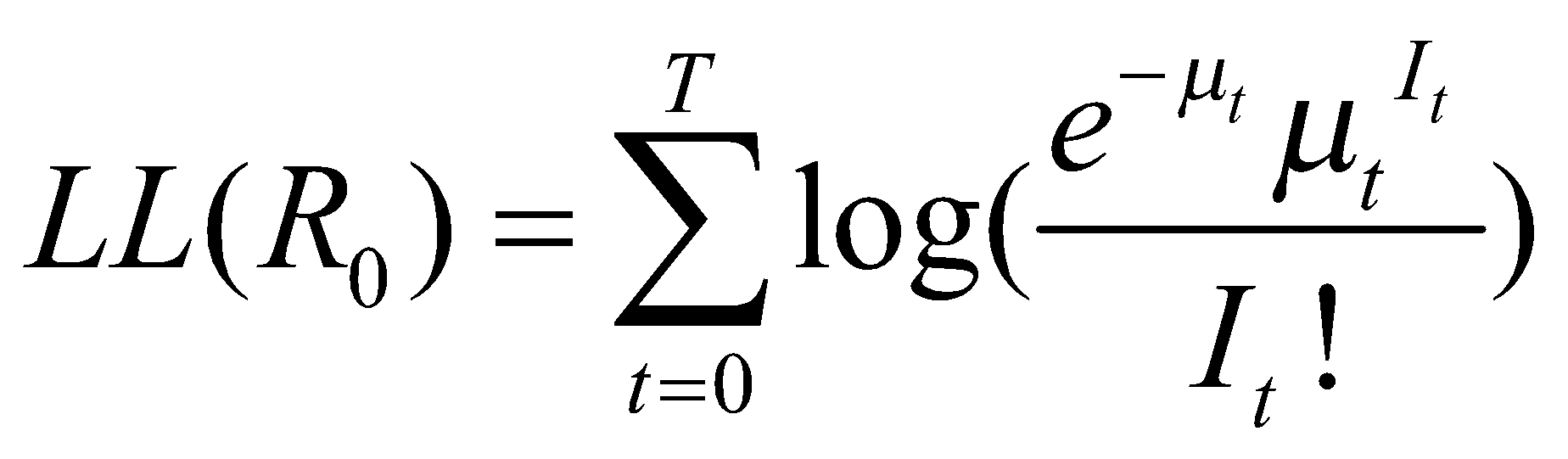
_

[2]

_
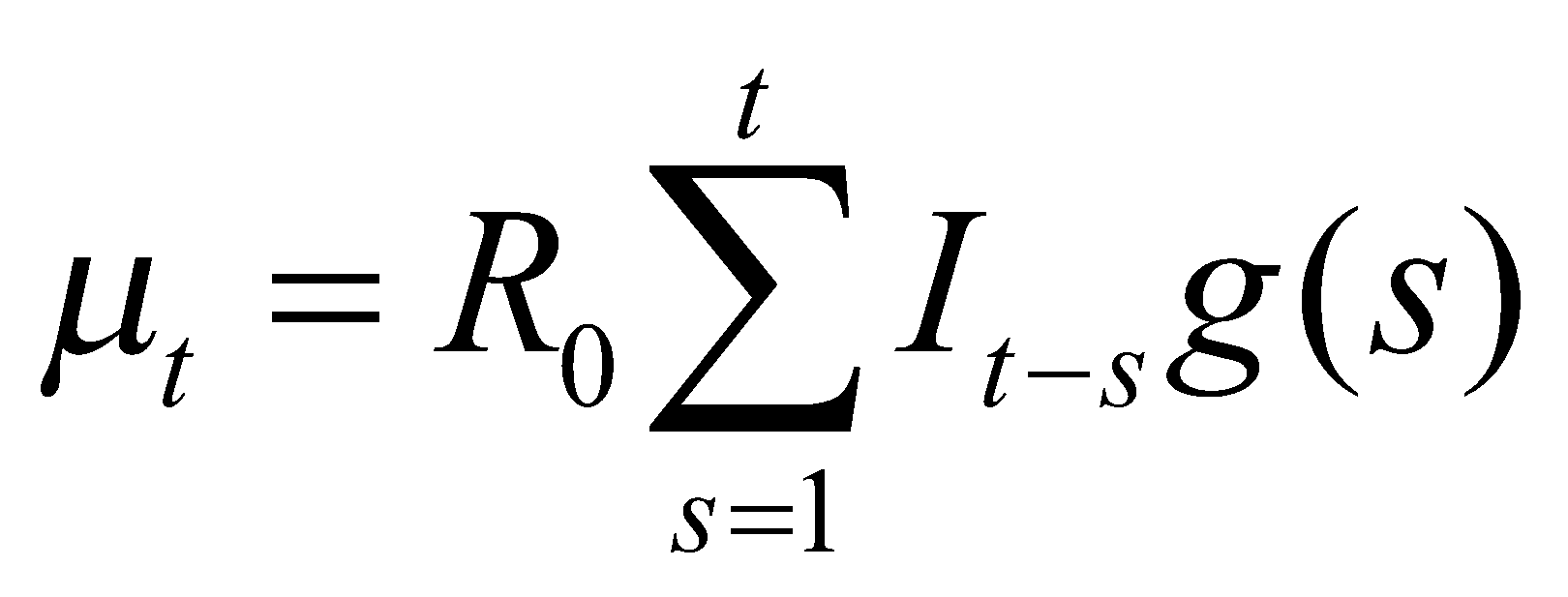
_

[3]

This method assumes an infinite number of susceptible individuals, with no importation or exportation of cases, and that the number of secondary cases generated by an index case is Poisson distributed (11).


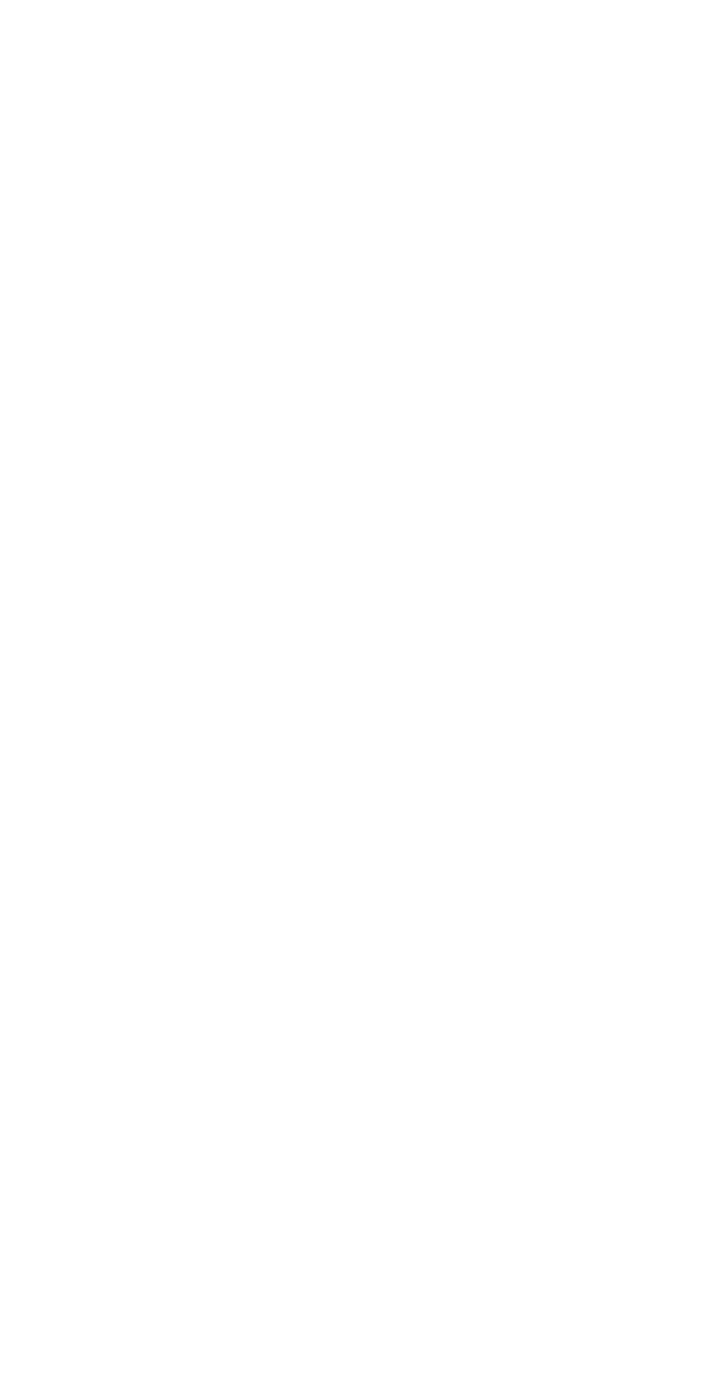


***3. EpiEstim method***

EpiEstim was originally designed to estimate time-varying reproduction numbers, R_t_, but can also be applied to estimate what can be interpreted as the basic reproduction number, R_0_ (12). As in the White and Pagano method, the likelihood assumes that case incidence is Poisson distributed with mean *µ_t_*, as shown in Equations 2 and 3. The posterior distribution of R_0_ is calculated in a Bayesian framework with a gamma distributed prior for R_0_ , with mean of 5 and standard deviation of 5 (12). Here the generation time distribution is used as an approximation for the infectivity profile of cases, i.e. the probability of an infected individual generating another case. Average R_0_ estimates were obtained by smoothing the time-dependent estimates across the period of interest, accounting for the generation time distribution. This method assumes no importation or exportation of cases. A recent update to this method has been developed to account for imported cases (28), however, we restrict our current analysis to that of closed systems.

***4. Wallinga and Teunis maximum likelihood method, with correction for yet-unobserved cases***

The Wallinga and Teunis method is also a time-dependent method which computes the reproduction number by averaging over all networks of transmission that are compatible with observed data (10). Here, the relative likelihood that case *i* has been infected by case *j*, normalized by the likelihood that case *i* has been infected by any other case *k* is:

_
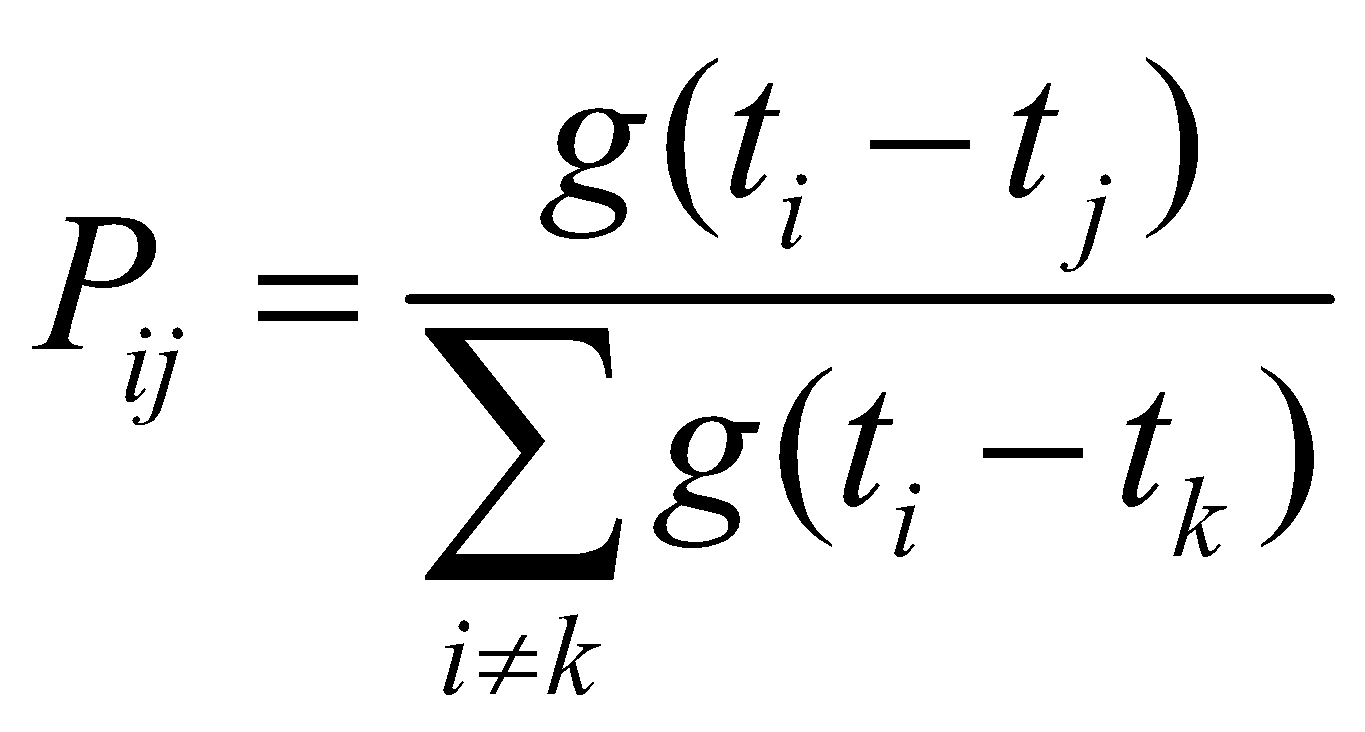
_

[4]

where *g* is the generation time distribution and *t_i_-t_j_* is the difference in time of symptom onset between cases *i* and *j*. Though the Wallinga and Teunis method is most often used to estimate the case reproduction number, which measures the reproduction number within a cohort of individuals with the same date of infection or symptom onset and accounts for past, present and future cases. In this analysis we apply the method only to cases observed at the current time point in order to assess real-time methodological performance. Average R_0_ estimates were obtained by smoothing the time-dependent estimates across the period of interest, which were corrected to account for secondary cases that are not yet observed (9).

***5. Bettencourt and Ribeiro method***

The Bettencourt and Ribeiro method is a real-time sequential Bayesian method which uses an approximation of the SIR model (31). The mean incidence at time t + 1, *I(t+1),* is assumed to be Poisson distributed as shown in equation 5, where γ^-1^ is the mean infectious period.

_
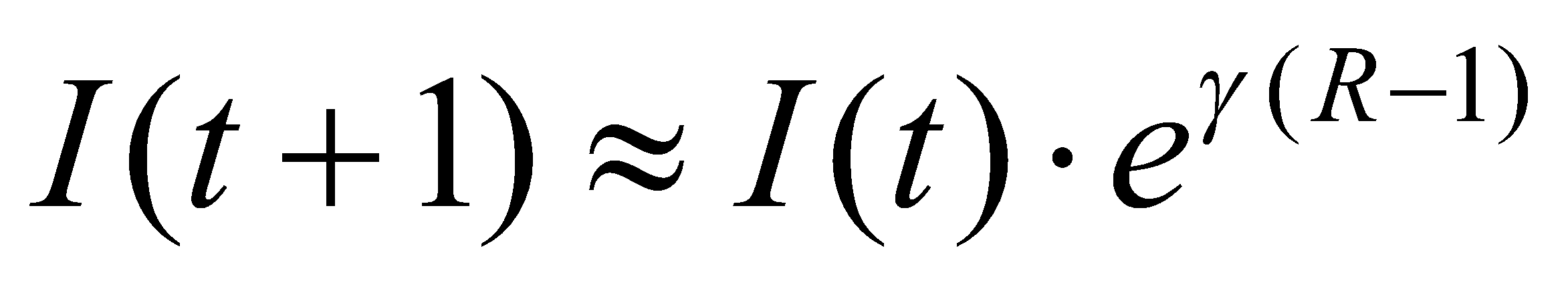
_

[5]

The algorithm begins its estimation with a non-informative prior distribution, which is updated sequentially so that the posterior distribution of the reproduction number at a given time is the prior distribution of the reproduction number at the next time point. Average R_0_ estimates were obtained by smoothing the time-dependent estimates across the period of interest, accounting for the generation time distribution.

*Supplementary Methods S2. Sensitivity analyses*

A sensitivity analysis was conducted to assess how mis-specification of the mean generation time would affect methodological performance. Methods were fit to the simulated Zika epidemics with no added noise where the mean generation time was mis-specified to be 20% greater and 20% less than the generation time used for simulations. Additional sensitivity analyses were conducted to assess how methodological performance might differ for pathogens with different generation time distributions to that of Zika. In the same manner as for the Zika epidemic simulations, 250 unmitigated epidemic simulations were generated using an ‘Ebola-like’ generation time distribution with a mean of 16 days and standard deviation of 9.3 days (assuming incubation and infectious periods of 11 and 5 days) and a ‘SARS-like’ generation time distribution with mean of 8 days and standard deviation 3.8 days (assuming incubation and infectious periods of 5 and 3 days). In line with weekly case reporting that occurred during the Zika epidemic, the mean generation time was approximated to the nearest number of weeks (i.e. ~3 weeks for Zika, ~2 weeks for Ebola and ~1 week for SARS) and fitted to case data approximating to the first 2, 3, 4 and 5 generations times. For the ‘Ebola-like’ generation time, this was approximated to the first 4, 6, 8 and 10 weeks of data and to the first 2, 3, 4 and 5 weeks for ‘SARS-like’ generation time.


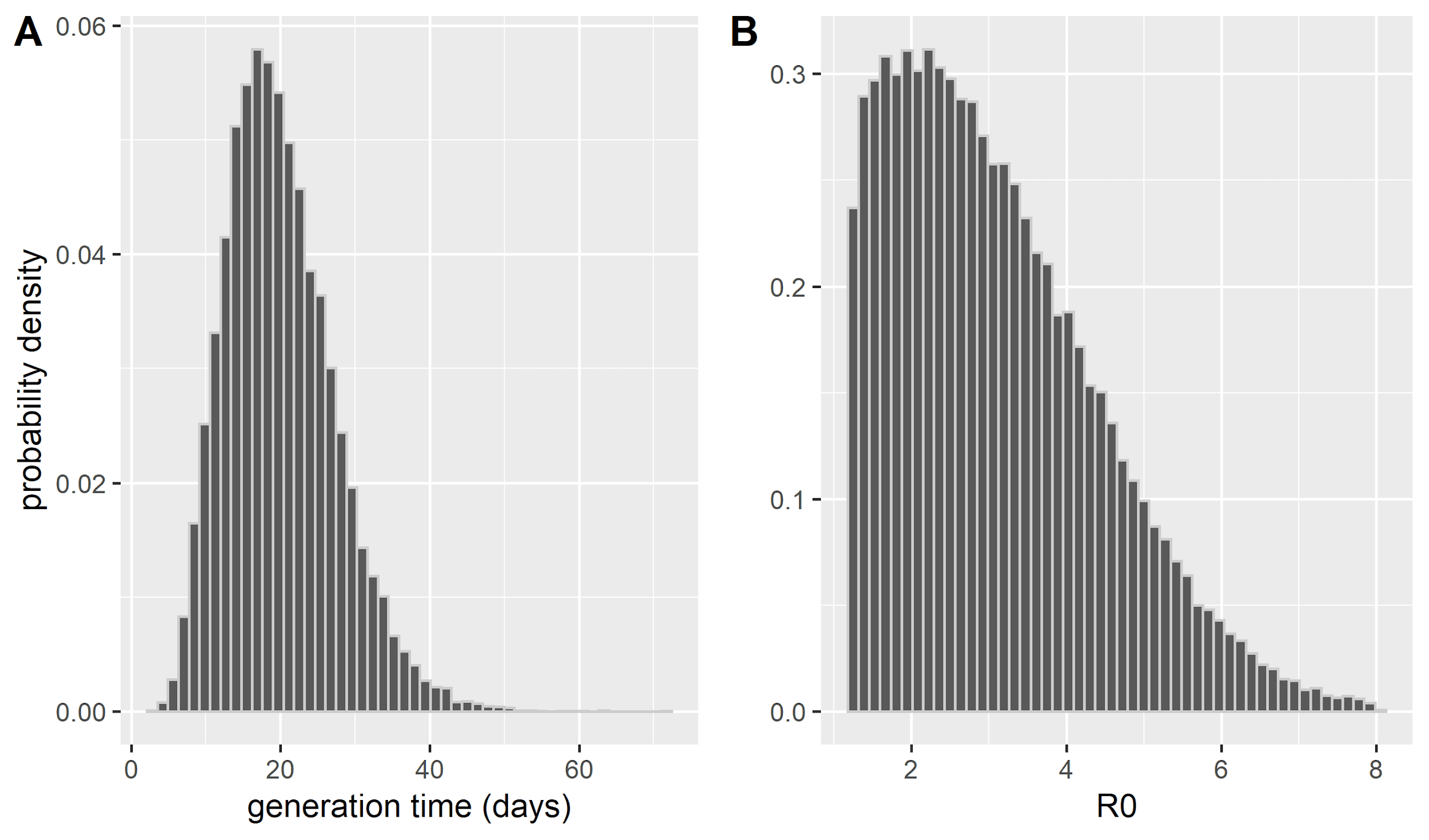


**Figure S1. Distribution of the generation time and R_0_ values used to simulate epidemic data.** Panel A shows the generation time distribution assumed in this analysis (gamma distribution with mean of 20 and standard deviation 7.4 days). Panel B shows the distribution of R_0_ values that was randomly drawn from for epidemic simulation (truncated normal distribution with mean of 2, standard deviation of 2, truncated between 1.2 and 8 days).


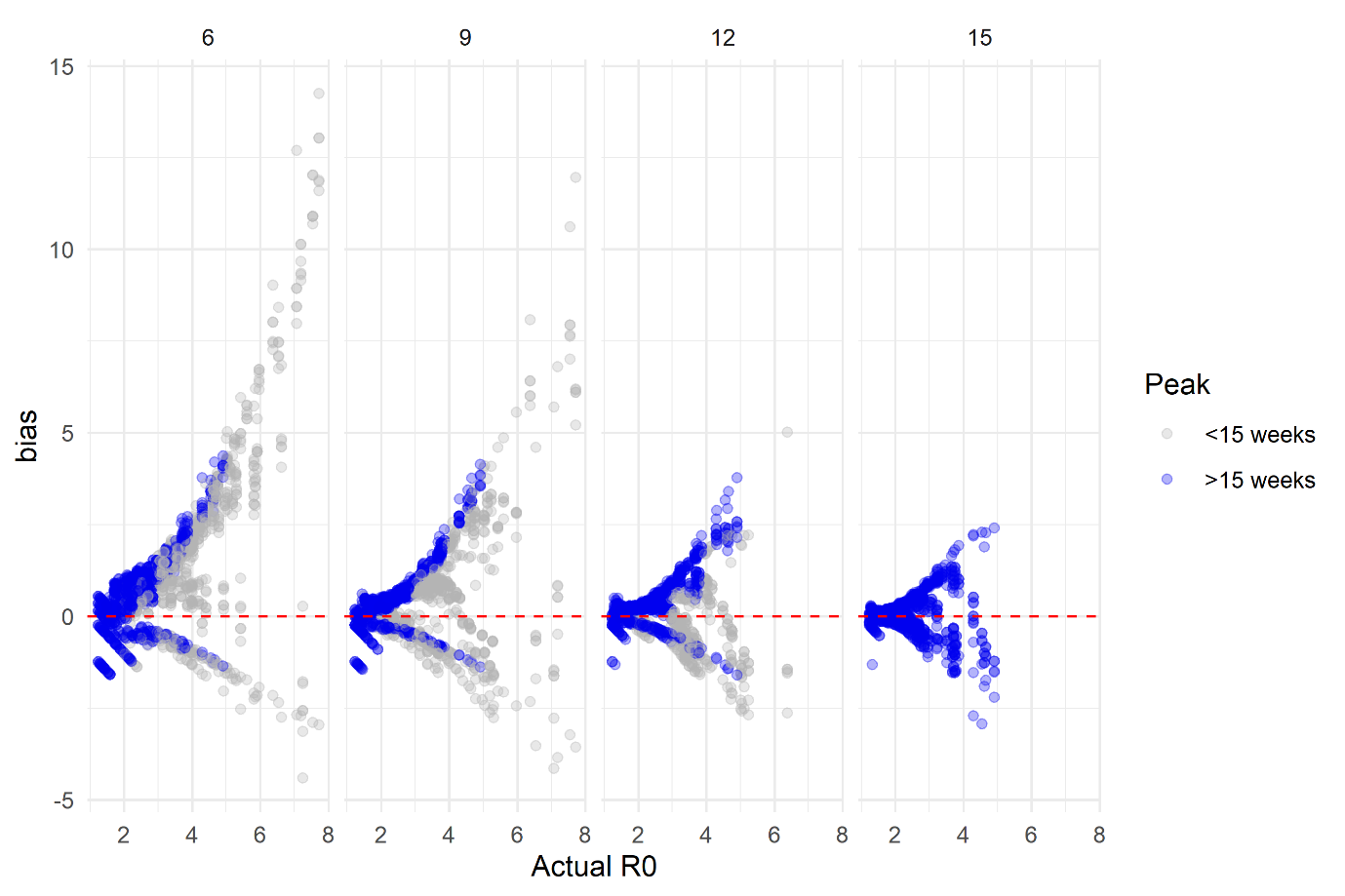


**Figure S2. Distribution of bias in R_0_ estimates (estimated R_0_ – actual R_0_) when fitting to an increasing number of data points in the case time series of simulated datasets (N=246) without noise, pooled across methods.** Columns represent the number of weeks fitted to in the case time series (6,9,12,15 weeks approximating to 2,3,4,5 generation times). Blue coloured points show the bias in estimates from simulations which peaked at or after 15 weeks (N=145), i.e. highlighting values with a consistent distribution of true R_0_ values across the 4 time points assessed. Grey points show bias in estimates from simulations that peaked earlier than 15 weeks, therefore not represented across all time points assessed here. Red dashed lines indicate a bias value of 0, i.e. estimated R_0_ = actual R_0_.


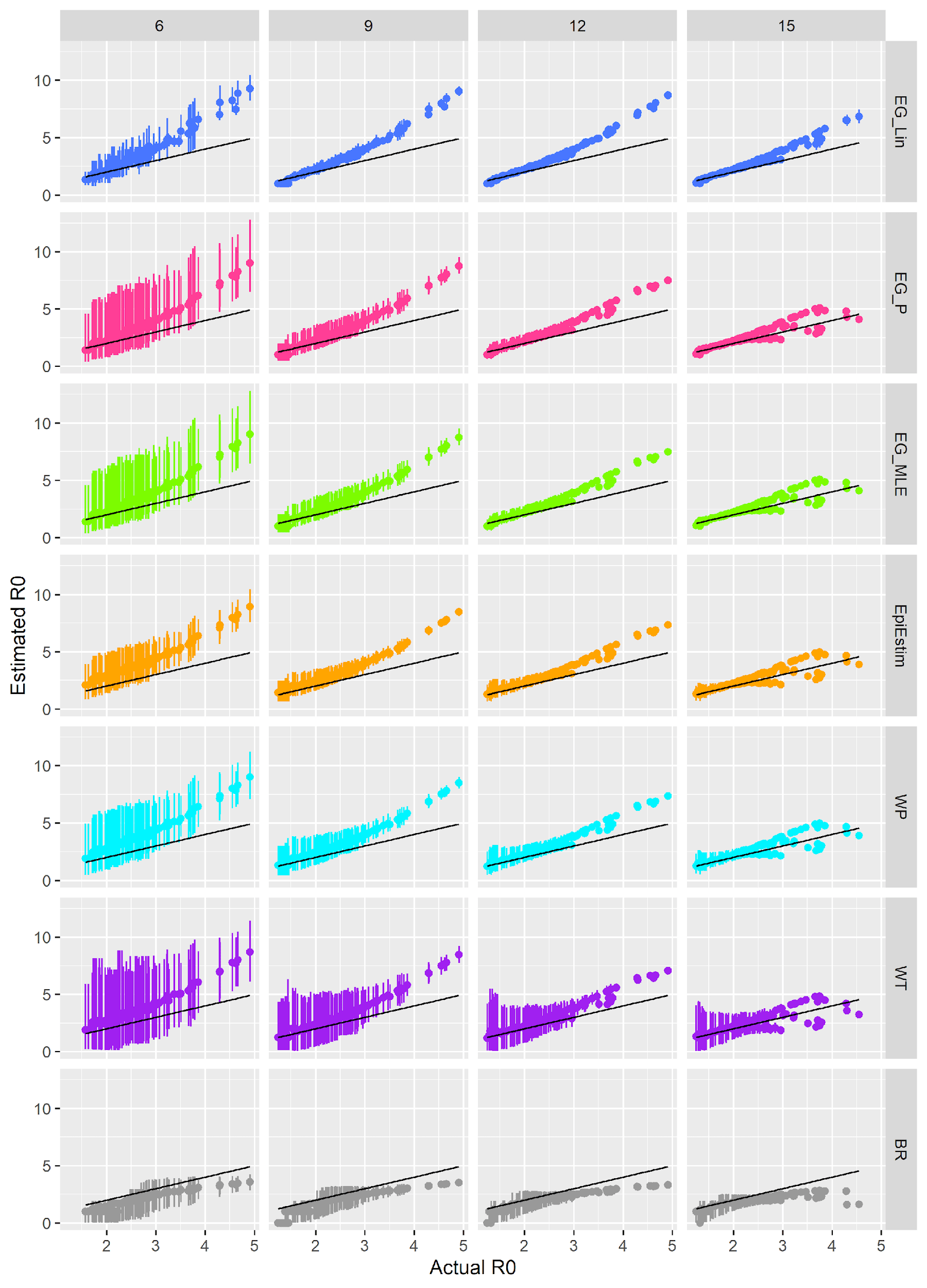


**Figure S3. Comparison of estimated and actual R_0_ values from 145 simulated datasets that peaked at or after 15 weeks, with no added noise.** Columns show the number of weekly data each method was fitted to, and colours represent the method. Black lines represent the y=x line. Method abbreviations: Linear exponential growth rate method (EG_Lin); Poisson exponential growth rate method (EG_P); maximum likelihood exponential growth rate method (EG_MLE); White and Pagano method (WP); Wallinga and Teunis method (WT); Bettencourt and Ribeiro (BR).


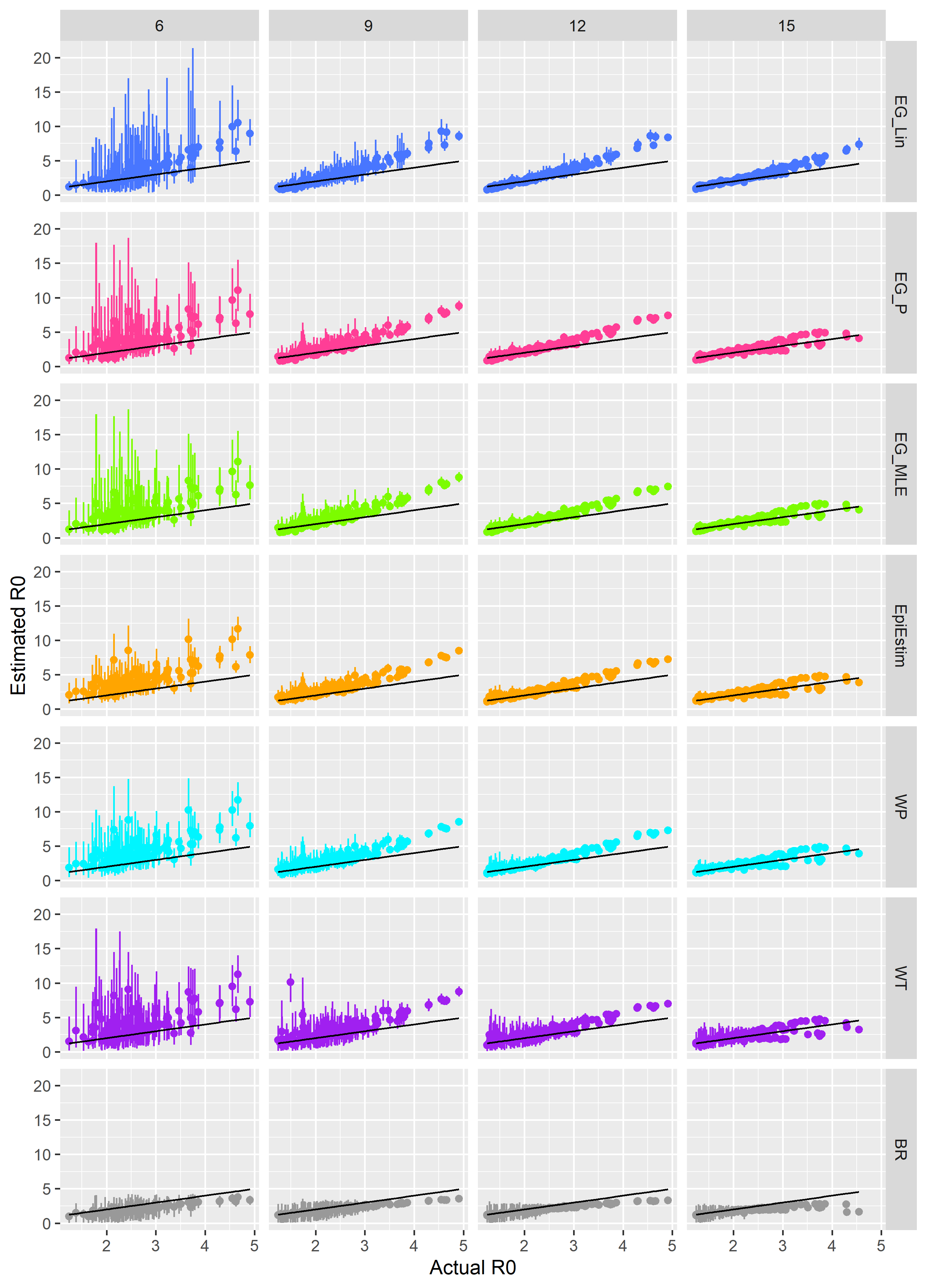


**Figure S4. Comparison of estimated and actual R_0_ values from 149 simulated datasets that peaked ≥15 weeks, with Poisson random errors.** Columns show the number of weekly data each method was fitted to, and colours represent the method. Black lines represent the y=x line. Method abbreviations: Linear exponential growth rate method (EG_Lin); Poisson exponential growth rate method (EG_P); maximum likelihood exponential growth rate method (EG_MLE); White and Pagano method (WP); Wallinga and Teunis method (WT); Bettencourt and Ribeiro (BR).


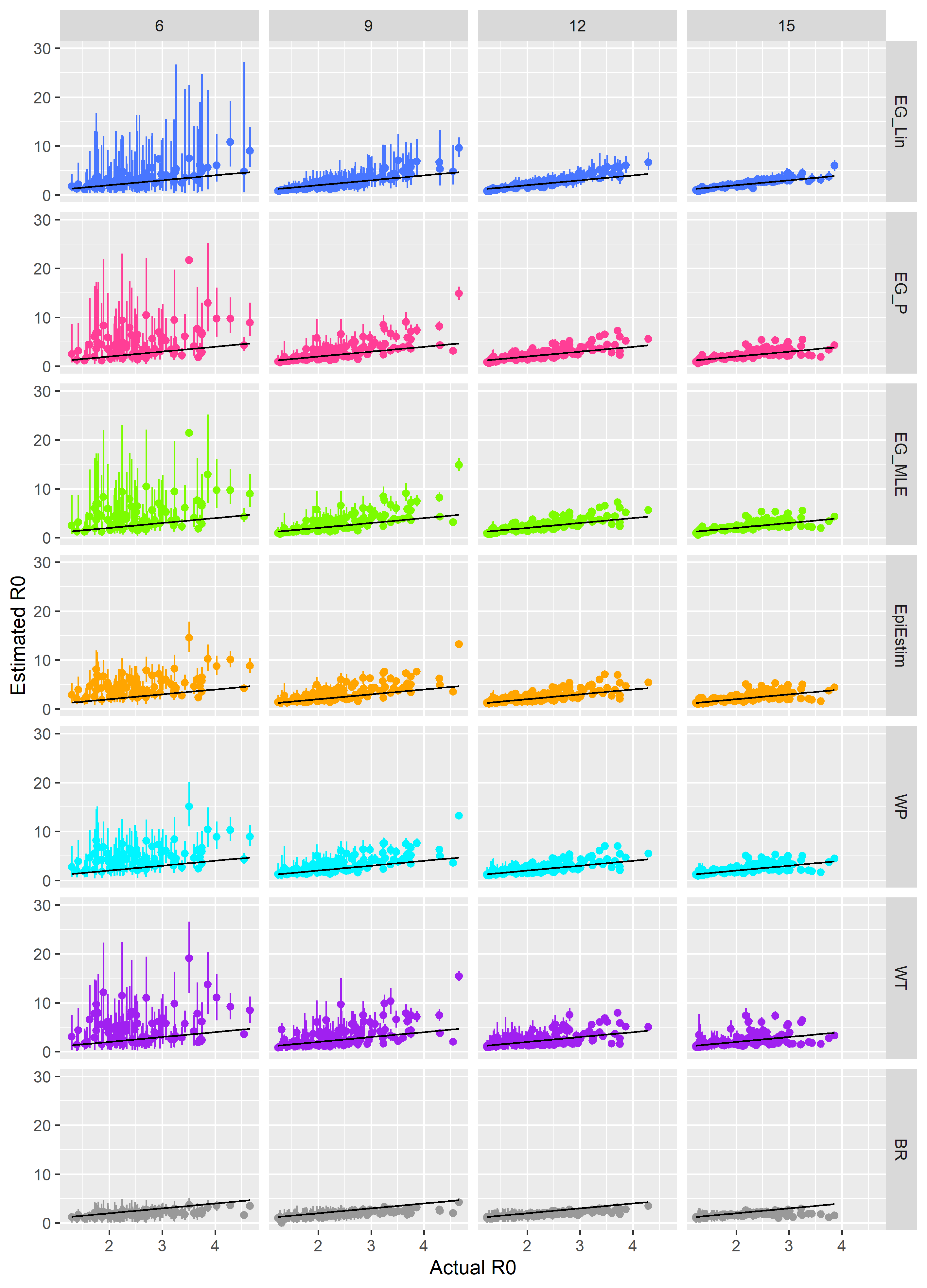


**Figure S5. Comparison of estimated and actual R_0_ values from 141 simulated datasets that peaked ≥15 weeks, with negative binomial random errors.** Columns show the number of weekly data each method was fitted to, and colours represent the method. Black lines represent the y=x line. Method abbreviations: Linear exponential growth rate method (EG_Lin); Poisson exponential growth rate method (EG_P); maximum likelihood exponential growth rate method (EG_MLE); White and Pagano method (WP); Wallinga and Teunis method (WT); Bettencourt and Ribeiro (BR).


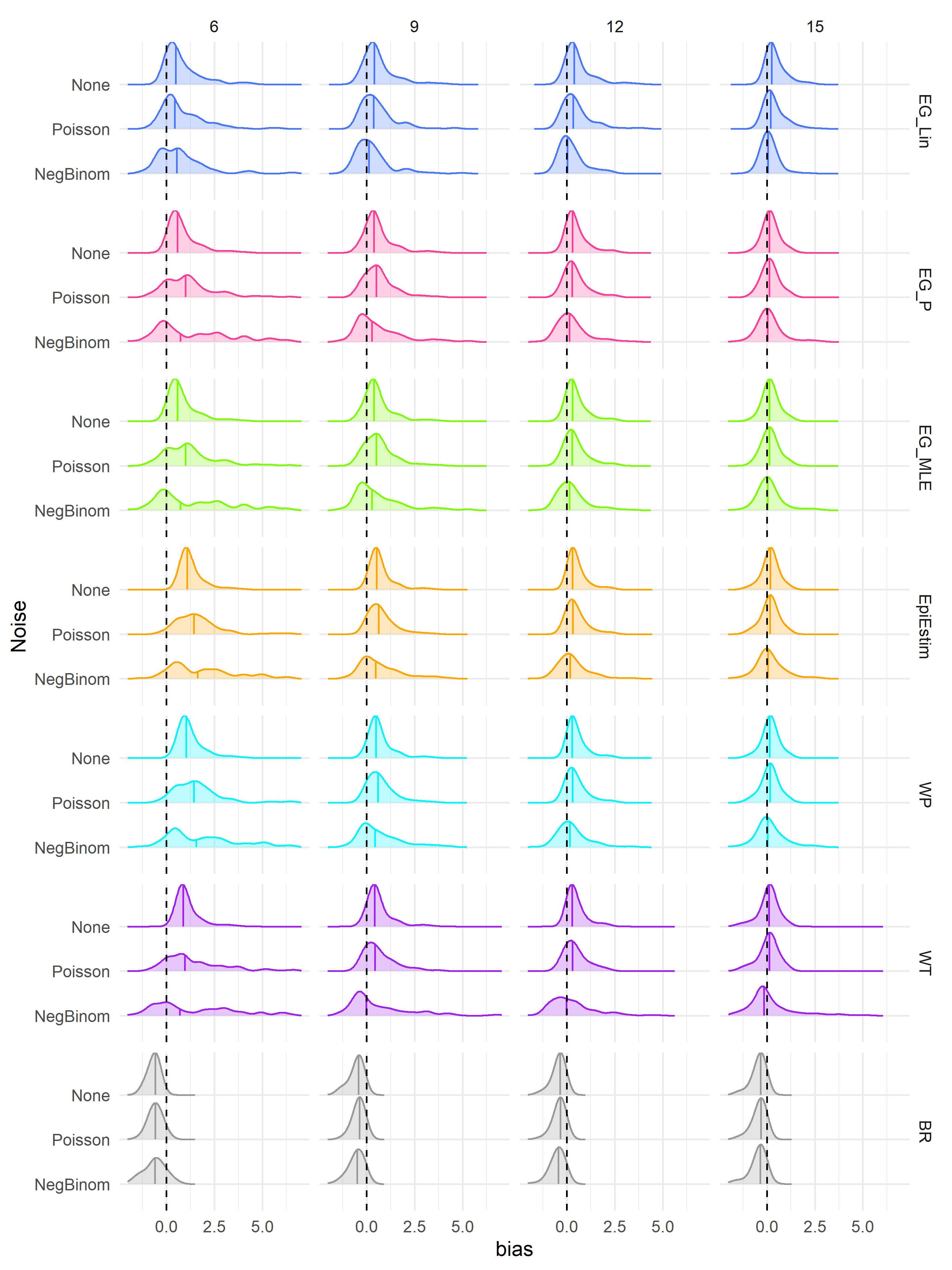


**Figure S6.** **Density distributions of bias in R_0_ estimates (estimated R_0_ – actual R_0_) obtained when fitting to the case time series of simulated data, by method, time point (in approximate generations), and noise scenario, using only results from simulations that peaked ≥15 weeks (N=146).** Columns represent the number of datapoints (weeks) each method was fitted to in the case time series (6,9,12,15 weeks). Black dashed lines highlight the ideal bias value of zero and coloured lines represent method-specific values of median bias. Method abbreviations: Linear exponential growth rate method (EG_Lin); Poisson exponential growth rate method (EG_P); maximum likelihood exponential growth rate method (EG_MLE); White and Pagano method (WP); Wallinga and Teunis (WT); Bettencourt and Ribeiro (BR).


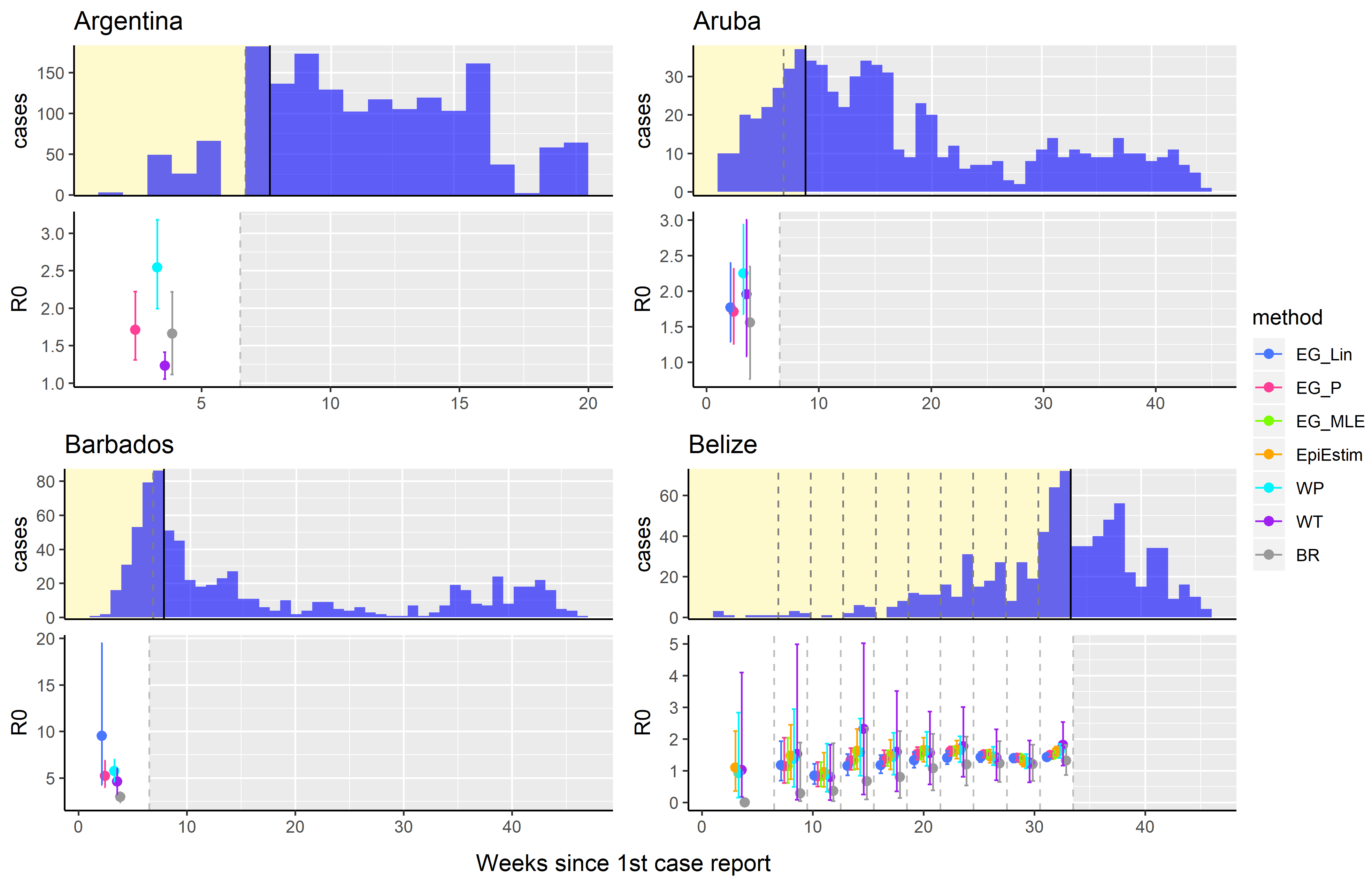


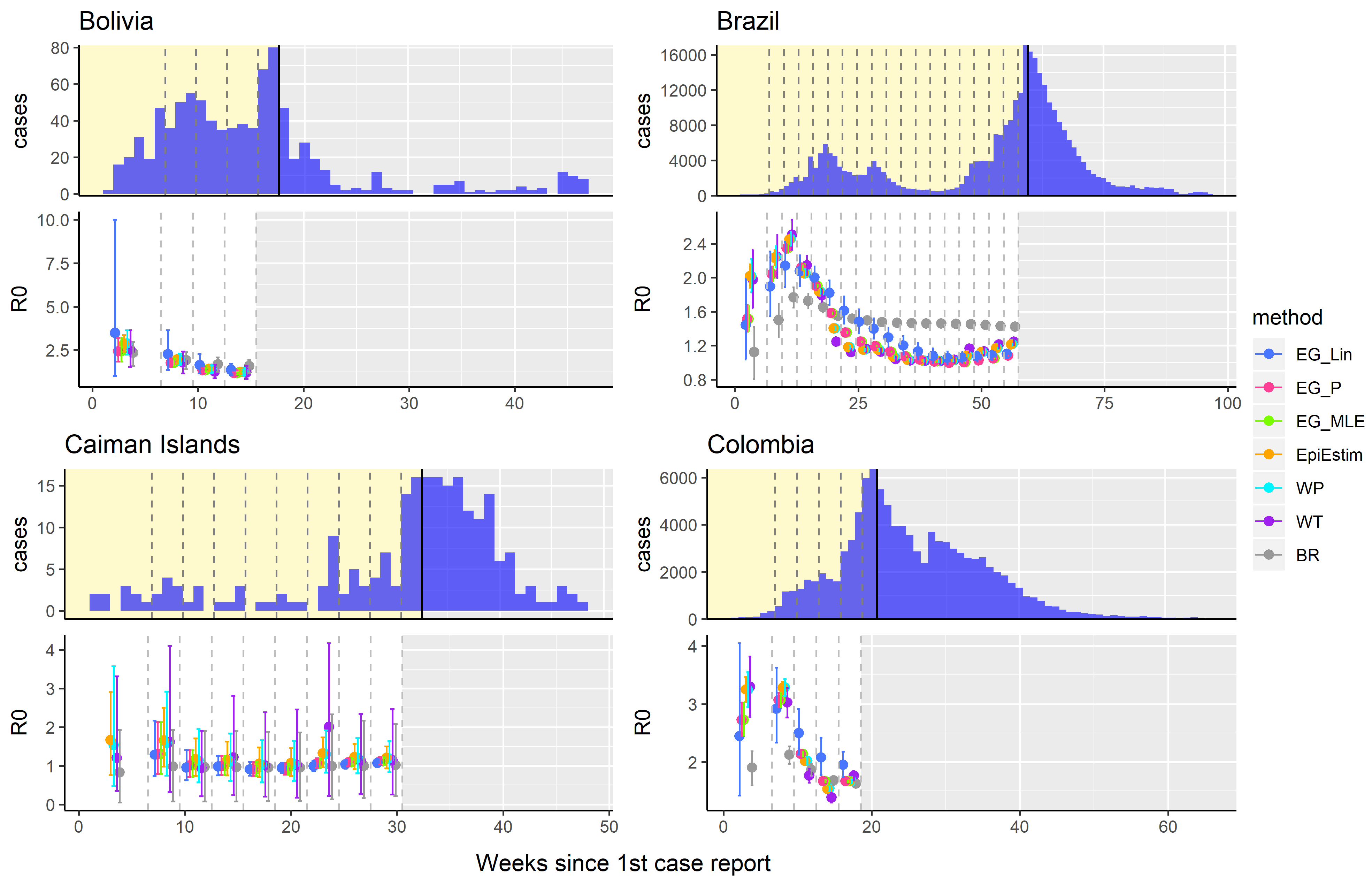


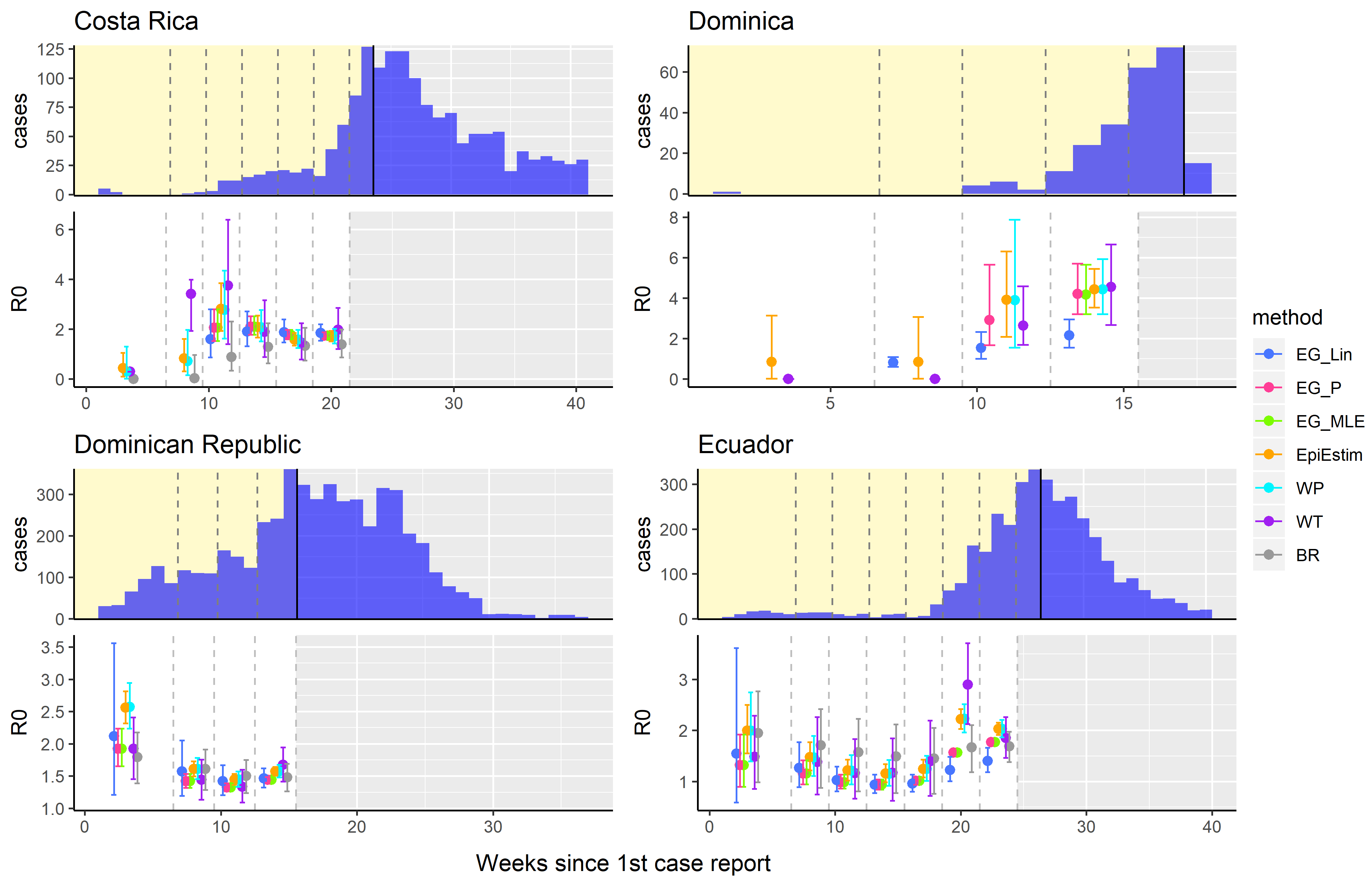


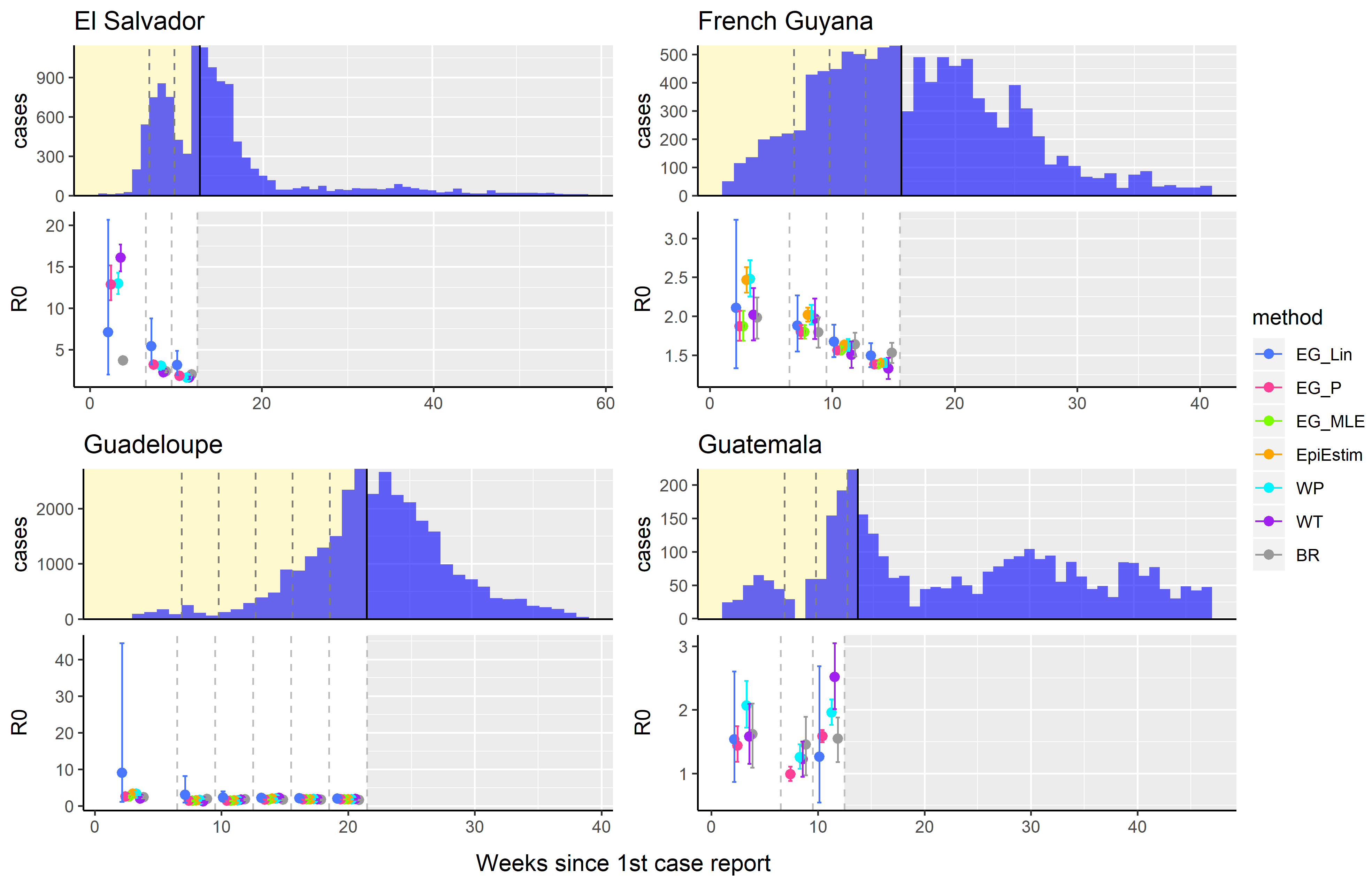


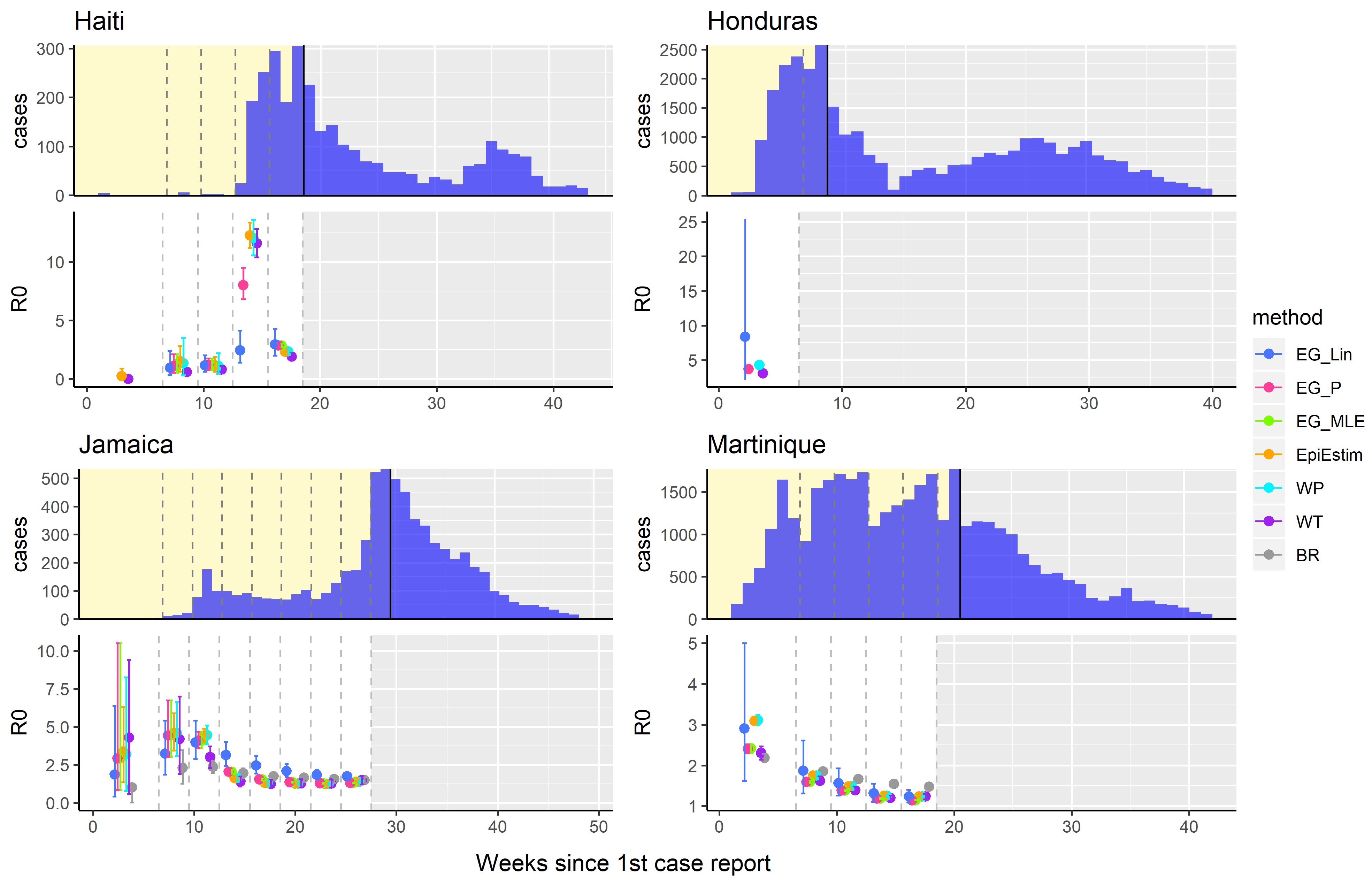


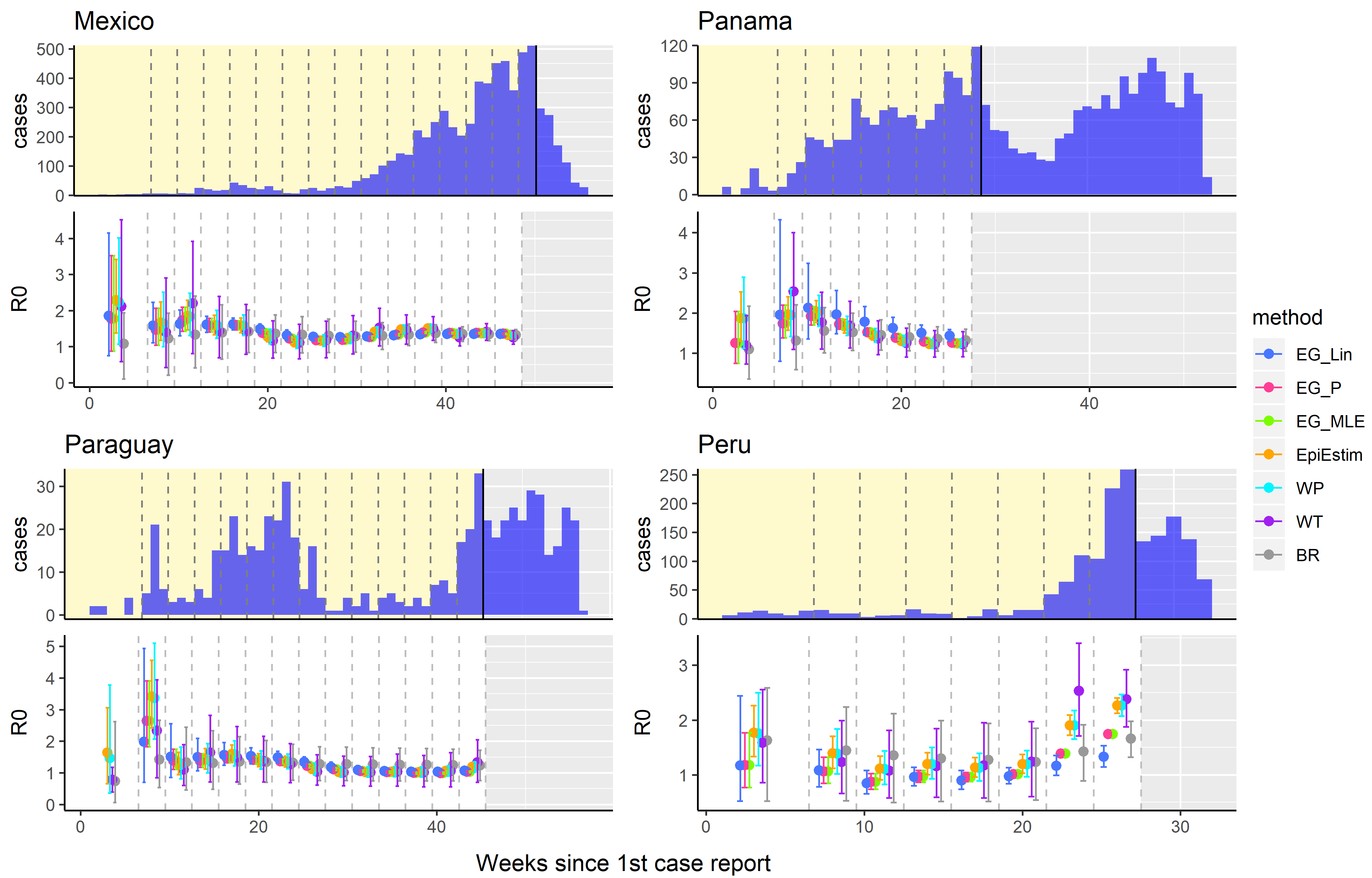


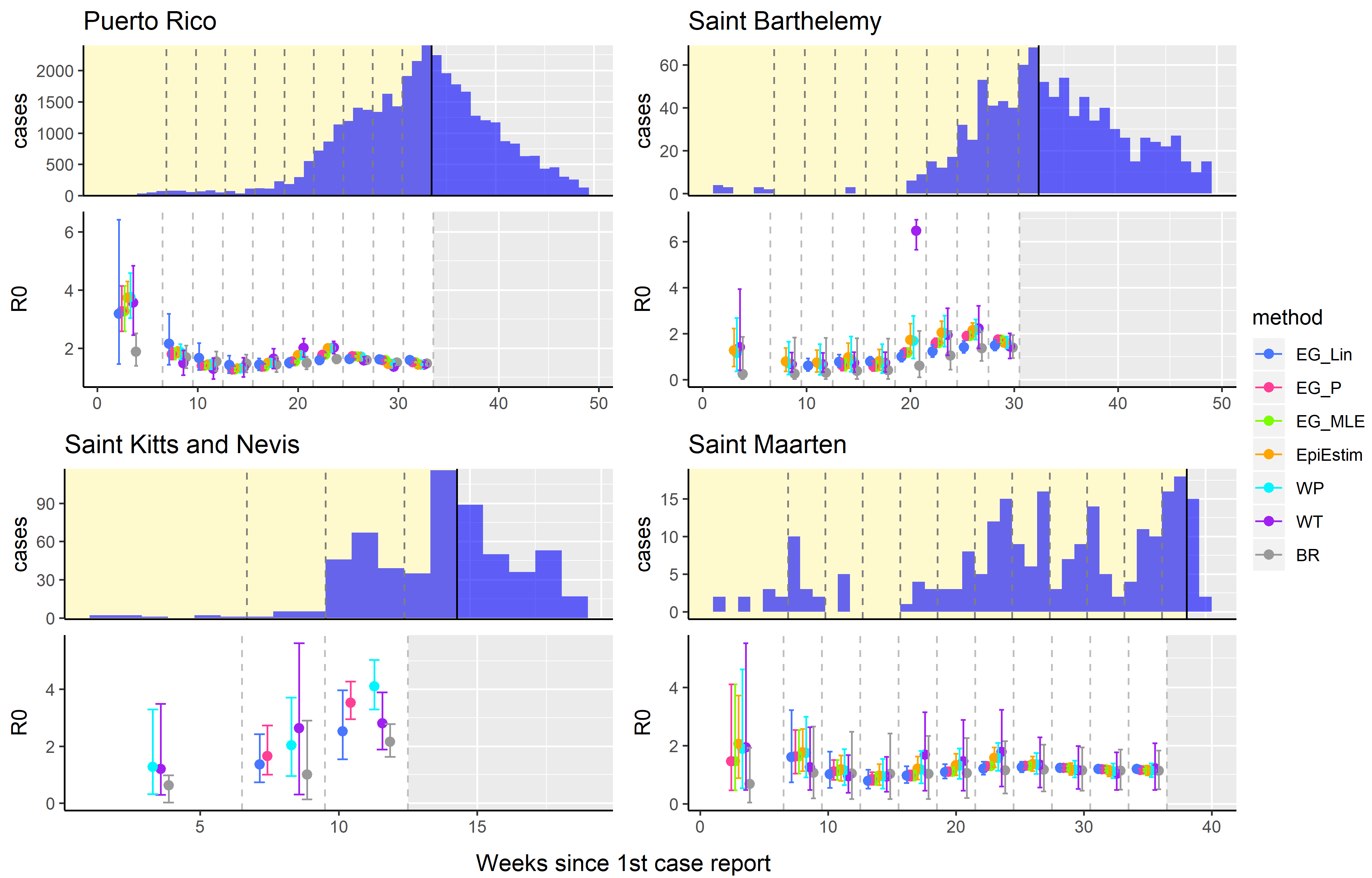


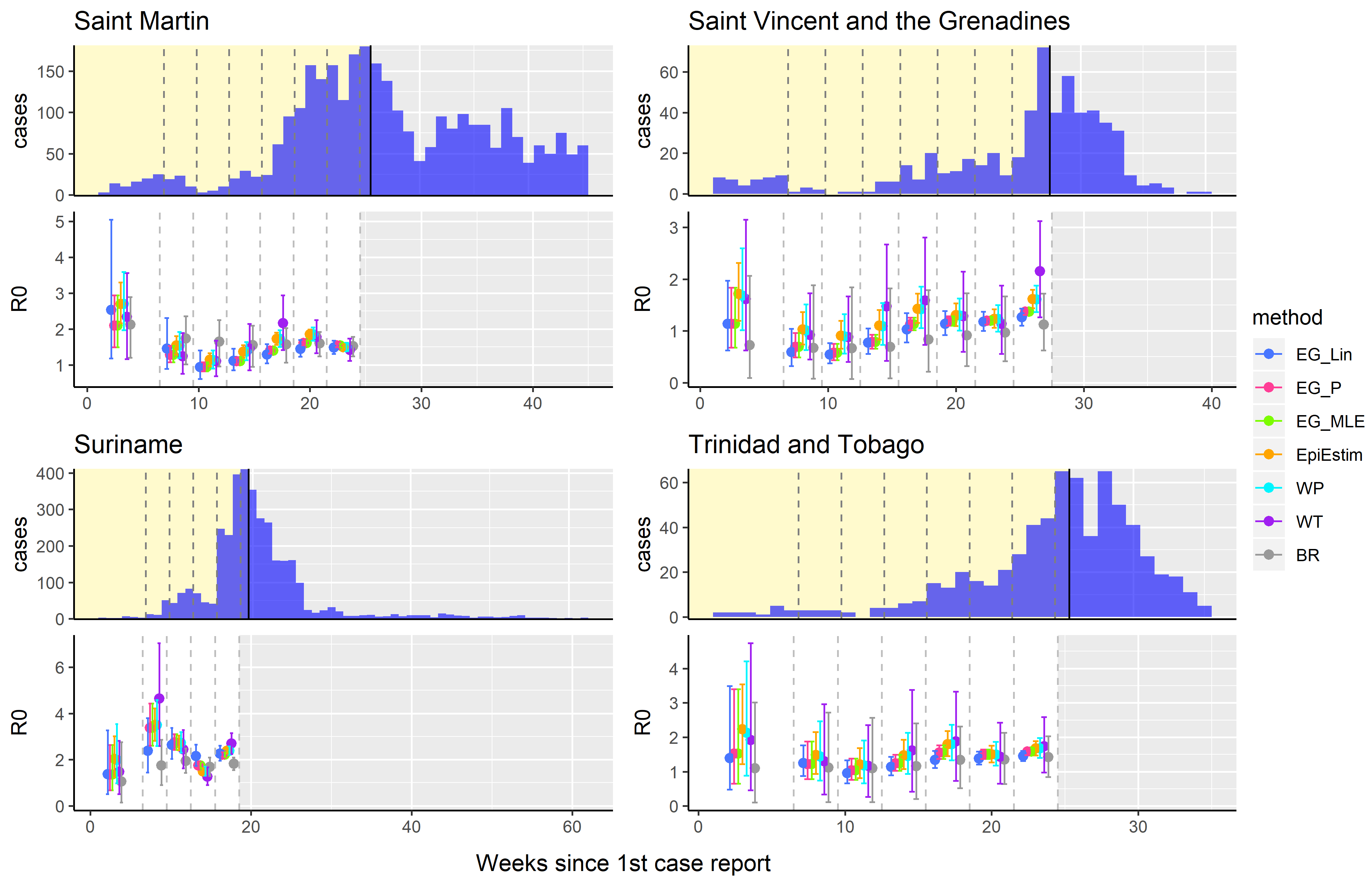


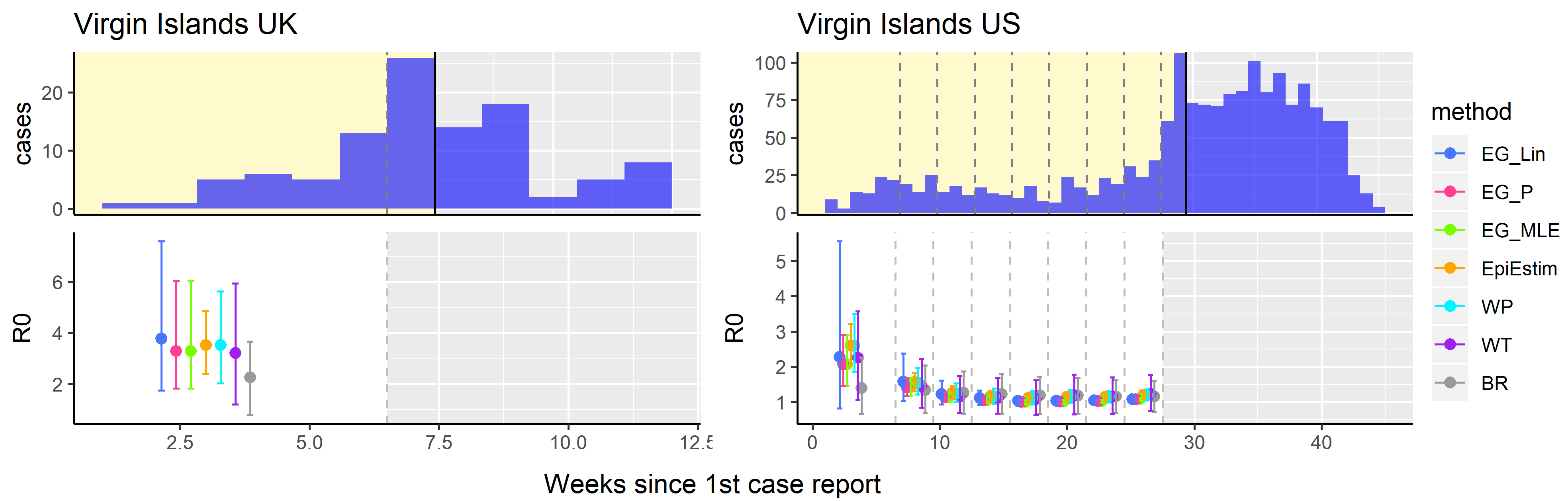


**Figure S7. R_0_ estimates obtained from each of the six methods fitted at different stages of the epidemic growth phase from the 2015-2016 Zika epidemics in Latin America and the Caribbean**. The top panel for each country shows the time series of reported Zika cases, with dashed lines showing the different stages at which each method was fitted to the data (first 6, 9, 12, etc., weeks) up to the peak of the epidemic, marked by the black line. The bottom panel for each country shows the mean and 95% confidence intervals of the R_0_ estimates produced with each method fitted to each time series. Method abbreviations: Linear exponential growth rate method (EG_Lin); Poisson exponential growth rate method (EG_P); maximum likelihood exponential growth rate method (EG_MLE); White and Pagano method (WP), Wallinga and Teunis (WT); Bettencourt and Ribeiro (BR).


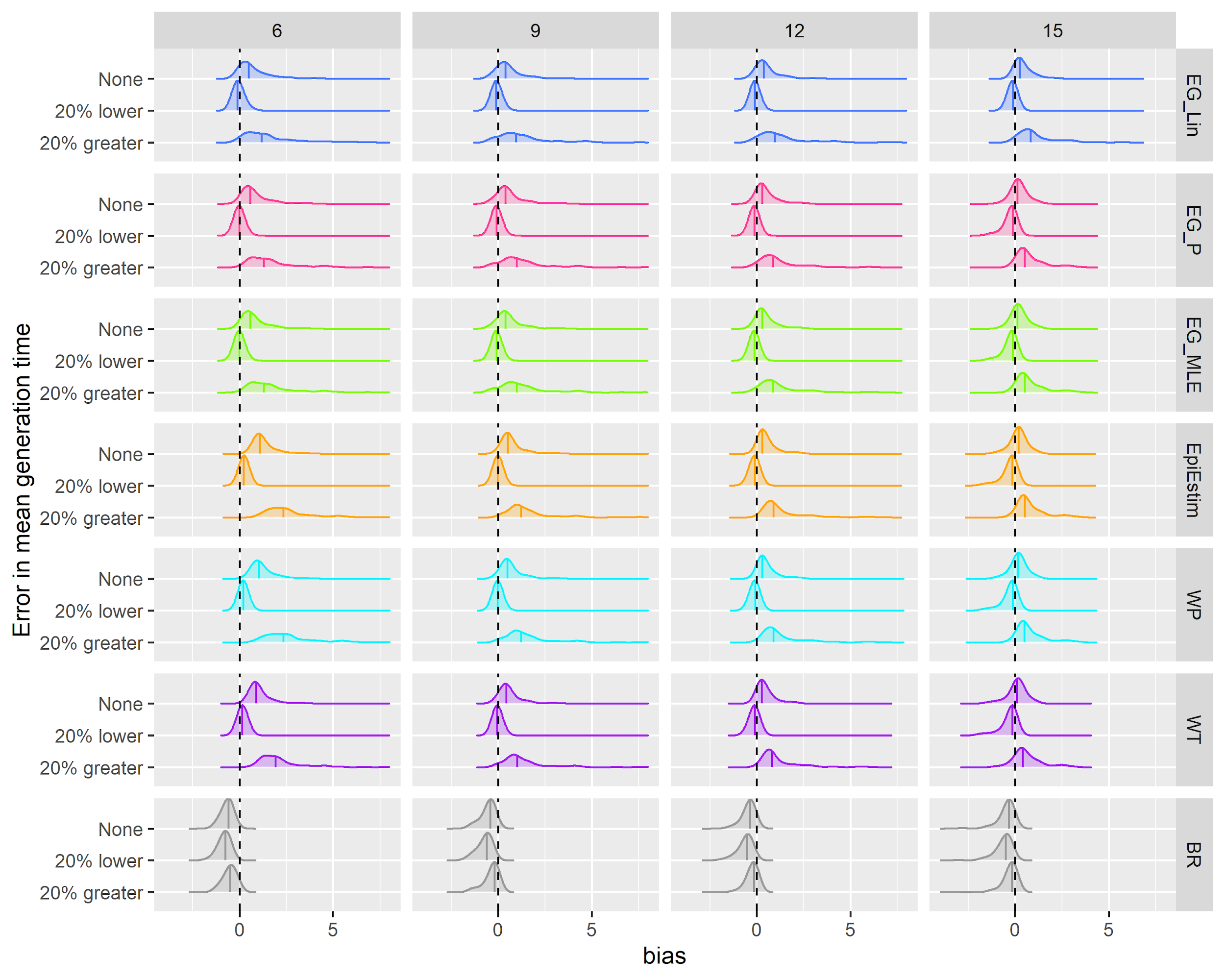


**Figure S8.** **Density distributions of bias in R_0_ estimates (estimated R_0_ – actual R_0_) obtained when fitting to the case time series of simulated data with variable specification of the mean generation time, by method, time point (number of weeks), on simulations with no added noise.** We considered scenarios where the mean generation interval was mis-specified as 20% greater than and 20% less than the actual mean generation time. Columns represent the number of datapoints (weeks) each method was fitted to in the case time series (6,9,12,15 weeks). Black dashed lines highlight the ideal bias value of zero and coloured lines represent method-specific values of median bias. Method abbreviations: Linear exponential growth rate method (EG_Lin); Poisson exponential growth rate method (EG_P); maximum likelihood exponential growth rate method (EG_MLE); White and Pagano method (WP); Wallinga and Teunis (WT); Bettencourt and Ribeiro (BR).


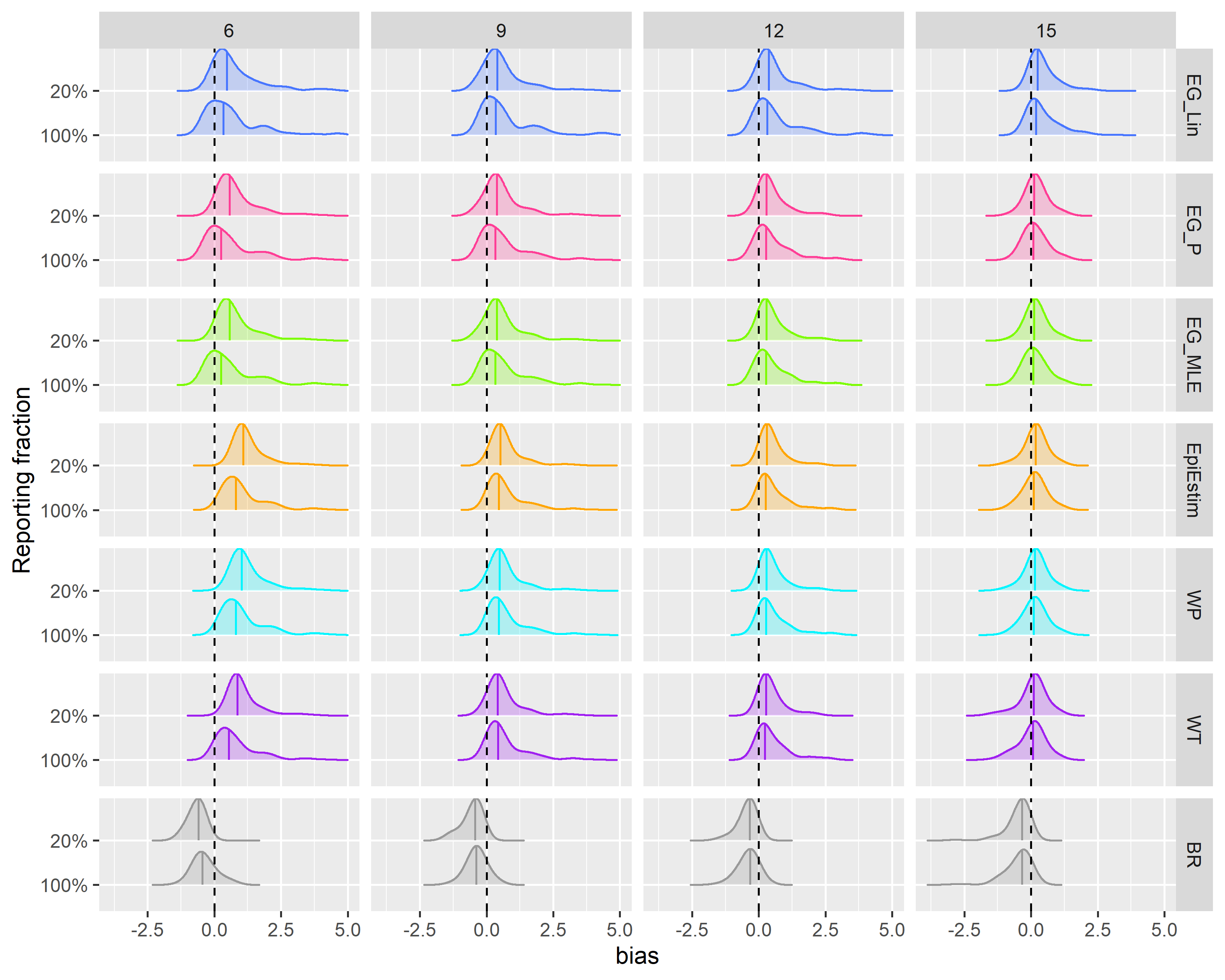


**Figure S9.** **Density distributions of bias in R_0_ estimates (estimated R_0_ – actual R_0_) obtained when fitting to the case time series of simulated data with different assumed reporting fractions, by method, time point (number of weeks), on simulations with no added noise.** We considered scenarios where 20% and 100% of infections resulted in reported cases which we assumed to be constant in time. Columns represent the number of datapoints (weeks) each method was fitted to in the case time series (6,9,12,15 weeks). Black dashed lines highlight the ideal bias value of zero and coloured lines represent method-specific values of median bias. Method abbreviations: Linear exponential growth rate method (EG_Lin); Poisson exponential growth rate method (EG_P); maximum likelihood exponential growth rate method (EG_MLE); White and Pagano method (WP); Wallinga and Teunis (WT); Bettencourt and Ribeiro (BR).

**References**

29. Obadia T, Haneef R, Boëlle PY. The R0 package: a toolbox to estimate reproduction numbers for epidemic outbreaks. BMC Med Inform Decis Mak **2012**; 12:147.

30. Wallinga J, Lipsitch M. How generation intervals shape the relationship between growth rates and reproductive numbers. Proc R Soc B Biol Sci **2007**; 274:599–604.

31. Bettencourt LMA, Ribeiro RM. Real time Bayesian estimation of the epidemic potential of emerging infectious diseases. PLOS One **2008**; 3:e2185.
